# Loss of function *CHCHD10* mutations in cytoplasmic TDP-43 accumulation and synaptic integrity

**DOI:** 10.1038/ncomms15558

**Published:** 2017-06-06

**Authors:** Jung-A. A. Woo, Tian Liu, Courtney Trotter, Cenxiao C. Fang, Emillio De Narvaez, Patrick LePochat, Drew Maslar, Anusha Bukhari, Xingyu Zhao, Andrew Deonarine, Sandy D. Westerheide, David E. Kang

**Affiliations:** 1 USF Health Byrd Alzheimer's Institute, University of South Florida, Morsani College of Medicine, Tampa, Florida 33613, USA; 2 Department of Molecular Medicine, University of South Florida, Morsani College of Medicine, Tampa, Florida 33613, USA; 3 Department of Cell Biology, Microbiology & Molecular Biology, University of South Florida, College of Arts and Sciences, Tampa, Florida 33620, USA; 4 James A. Haley Veteran's Administration Hospital, Tampa, Florida 33612, USA

## Abstract

Although multiple *CHCHD10* mutations are associated with the spectrum of familial and sporadic frontotemporal dementia–amyotrophic lateral sclerosis (FTD–ALS) diseases, neither the normal function of endogenous CHCHD10 nor its role in the pathological milieu (that is, TDP-43 pathology) of FTD/ALS have been investigated. In this study, we made a series of observations utilizing *Caenorhabditis elegans* models, mammalian cell lines, primary neurons and mouse brains, demonstrating that CHCHD10 normally exerts a protective role in mitochondrial and synaptic integrity as well as in the retention of nuclear TDP-43, whereas FTD/ALS-associated mutations (R15L and S59L) exhibit loss of function phenotypes in *C. elegans* genetic complementation assays and dominant negative activities in mammalian systems, resulting in mitochondrial/synaptic damage and cytoplasmic TDP-43 accumulation. As such, our results provide a pathological link between CHCHD10-associated mitochondrial/synaptic dysfunction and cytoplasmic TDP-43 inclusions.

## Abstract (web summary)

Mutations in *CHCHD10* have been recently associated with frontotemporal dementia and amyotrophic lateral sclerosis. Here the authors study the functions of endogenous CHCHD10 in *Caenorhabditis elegans*, primary neurons, and mouse, and show that it normally protects mitochondria and synaptic integrity, and retains TDP-43 in the nucleus.

Frontotemporal dementia (FTD) is a progressive neurodegenerative disease classically characterized by the selective degeneration of the frontal and temporal lobes, associated with executive impairments, changes in personality and language dysfunction. It is now clear, however, that FTD is frequently accompanied by motor neuron disease or amyotrophic lateral sclerosis (ALS), and common genetic mutations in multiple genes have been associated with both FTD and ALS^1^,^2^. Indeed, ∼15% of FTD patients also suffer from ALS, and up to 50% of classical ALS patients also suffer from symptoms of FTD or mild cognitive impairment^2^. The unifying pathological link between the vast majority of ALS and FTD patients is the accumulation of cytoplasmic TDP-43 inclusions, the latter in the majority FTD subtype, frontotemporal lobar degeneration with TDP-43 pathology (FTLD-TDP)^3^,^4^.

TAR DNA-binding protein 43 (TDP-43) is a nuclear protein in the family of heterogeneous ribonucleoproteins (hnRNPs) that plays a major role in regulating RNA splicing, stability and transport^5^. However, in pathological neurons, TDP-43 is often found in the cytoplasm in a ubiquitinated and fragmented form, which are prone to aggregation^6^,^7^,^8^. While TDP-43 mutations are found in a small proportion of ALS and FTLD-TDP, TDP-43 pathology is associated with the vast majority of ALS and FTLD^4^,^9^. Increasing evidence indicates that TDP-43 is highly neurotoxic, in large part, by inducing mitochondrial dysfunction^10^,^11^,^12^. Specifically, TDP-43 is co-localized with mitochondria and reduces mitochondrial length by promoting mitochondrial fission, resulting in mitochondrial transport defects^10^. The mitochondrial fusion protein Mfn2 prevents mitochondrial fragmentation, superoxide production and depolarization (ΔΨm) induced by TDP-43 mutations^10^, and blockade of TDP-43 translocation to mitochondria also blocks its neurotoxicity^12^. In line with these observations, TDP-43 overexpression in transgenic mice increases mitochondrial fission proteins Drp1 and Fis1, reduces the mitochondrial fusion protein Mfn1, and promotes mitochondrial fragmentation and aggregation^11^.

Coiled-coil-helix-coiled-coil-helix domain containing 10 (*CHCHD10*) encodes a protein that is localized to the intermembrane space of mitochondria^13^ associated with the mitochondrial contact site and cristae organizing system (MICOS) together with mitofusin, mitophilin, CHDHD3 and CHCHD6 (ref. 14). Recent human genetic studies have identified multiple *CHCHD10* mutations in sporadic and familial FTD–ALS spectrum disorders from independent cohorts and ethnic groups^13^,^14^,^15^,^16^,^17^, implicating the critical role of mitochondria in FTD–ALS diseases. *CHCHD10* mutations have also been associated with Charcot–Marie–Tooth disease type 2 (ref. 18), mitochondrial myopathy and spinal muscular atrophy Jokela type^19^. However, despite the role of the FTD/ALS-linked CHCHD10 S59L mutation in reducing mitochondrial length and disorganization of mitochondrial cristae morphology^13^, neither the normal function of endogenous CHCHD10 nor the nature of the FTD/ALS *CHCHD10* mutations has been investigated. Moreover, whether and how *CHCHD10* mutations alter the pathological landscape (that is, TDP-43 inclusions) of FTD–ALS spectrum diseases is completely unknown, and no FTD/ALS mutations other than S59L have been functionally characterized. Here we show in *Caenorhabditis elegans* models, cultured cells, primary neurons, and mouse brains that CHDHD10 normally plays a neuroprotective role and that FTD/ALS CHCHD10 mutations (R15L and S59L) display loss of function phenotypes and function in concert with TDP-43 to induce its cytoplasmic mislocalization, resulting in mitochondrial and synaptic damage.

## Results

### Loss of *har-1* loss impairs movement and mitochondria in *C. elegans*

**Figure 1:**
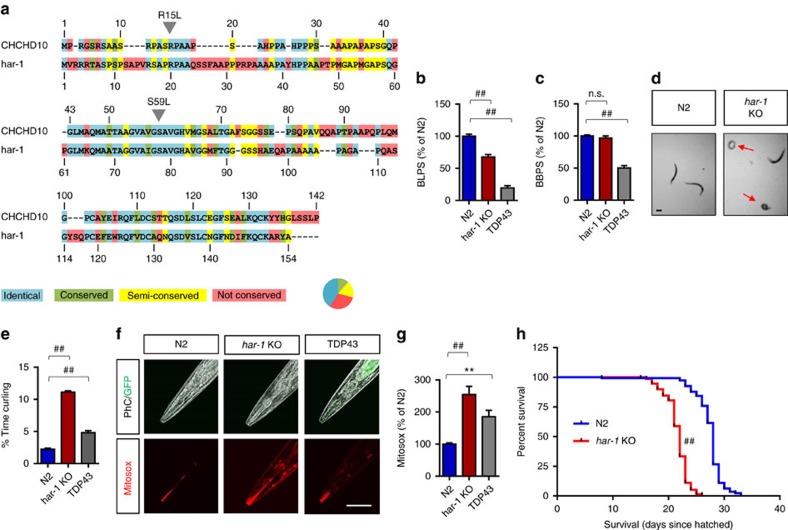
Loss of har-1 impairs locomotion mitochondrial health and longevity in *C. elegans*. (**a**) Protein sequence alignment between human CHCHD10 and *C. elegans* har-1. Note that *C. elegans* har-1 shares 41% identity, 12% conserved similarity, and 18% semi-conserved similarity to human CHCHD10 (http://useast.ensembl.org/index.html). (**b**–**e**) Age-synchronized worms per strain (3-day adults: N2, *har-1* KO (*har-1*^−/−^) and TDP43) transferred to fresh NGM plate or M9 buffer and videotaped to measure motility, liquid thrashing rate, and curling behaviour (1-way ANOVA, *post hoc* Tukey, ^##^*P*<0.0001). (**b**) Motility (measured in BLPS) on NGM plate measured at ambient room temperature (22 °C) and normalized to N2 control from 90 to 94 worms per strain. (**c**) Liquid thrashing rate (BBPS) measured in M9 buffer at ambient room temperature (22 °C) and normalized to N2 control from 50 worms per strain. (**d**,**e**) Representative images of curling posture (red arrows). Per cent time spent curling measured at ambient room temperature (22 °C) from 50 worms per strain (scale bar, 100 μm). (**f**,**g**) Age-synchronized worms stained with 5 μM mitosox-red in M9 buffer for 45 min, mounted live on agar pads, imaged by confocal microscopy, quantified with Image J, and normalized to N2 control (one-way ANOVA, **P<0.01, ^##^*P*<0.0001, *n*=29 worms/strain, scale bar, 100 μm). Average intensity of head region excluding mouth, pharynx, and intestine quantified. (**h**) Lifespan analysis (% of worms surviving since days hatching) of N2 and *har-1* KO worms at 16 °C (Log-rank Mantel–Cox test, ^##^*P*<0.0001, *n*=170 worms/strain). All quantitative graphs with error bars were expressed as mean±s.e.m. ANOVA, analysis of variance; KO, knockout.

The *C. elegans har-1* gene is the closest orthologue to mammalian *CHCHD10* with 41% identity, 12% conserved homology and 18% semi-conserved homology to human CHCHD10 at the level of amino acid sequence (Fig. 1a). The R15 and S59 CHCHD10 residues mutated in FTD/ALS are conserved between *C. elegans* har-1 and human CHCHD10 proteins (Fig. 1a). To gain insights to the function of CHCHD10 *in vivo*, we utilized the VC3169 *har-1*^−/−^ *C. elegans* model (*gk3124*) to assess locomotion in comparison with wild-type N2 ancestral and TDP-43 transgenic strains. TDP-43 expression in the TDP-43 transgenic *C. elegans* CL6049 is driven by the neuron-specific *snb-1* promoter, a strain with impairments in locomotion at room temperature that worsens with heat exposure^20^. In motility tests at ambient temperature, we observed significantly slower rates of movement in both *har-1*^−/−^ and TDP-43 transgenic worms by 32 and 80%, respectively, as compared to N2 (Fig. 1b). In liquid thrashing tests at ambient temperature, *har-1*^−/−^ worms showed no significant reduction in body bends per second (BBPS), whereas the TDP-43 worms displayed a significant 50% reduction in BBPS (Fig. 1c). Upon exposure to heat (30 °C for 30 min), both TDP-43 and *har-1*^−/−^ strains showed dramatically reduced liquid thrashing rates compared to N2 controls (Supplementary Fig. 1a), indicating that heat exposure renders the *har-1*^−/−^ strain more vulnerable to the loss of *har-1* possibly due to proteostatic stress. Next, we tested the strains for the percentage of time spent in a curled posture in liquid curling tests, since both TDP-43 and *har-1*^−/−^ worms showed salient abnormalities in their pattern of thrashing in liquid (Fig. 1d, Supplementary Fig. 1b and Supplementary Movies 1,2). Interestingly, abnormal curling behaviour increases with age in *C. elegans* and is reflective of a defect in motor coordination^21^. At ambient temperature, *har-1*^−/−^ and TDP-43 strains showed 4.9-fold and 2.1 increases in time spent in curled posture while thrashing in liquid, respectively (Fig. 1e). This differential phenotype was especially accentuated in the TDP-43 worms when exposed to 30 °C (Supplementary Fig. 1c). As har-1 is a mitochondrial protein^22^, we tested the hypothesis that mitochondrial dysfunction might underlie the loss of *har-1*. We therefore subjected the strains to mitosox-red staining, which indicates the level of mitochondrial superoxide, with higher levels of superoxide corresponding to poorer mitochondrial health. Quantification of mitosox-red intensity in the head region (excluding mouth, pharynx and intestines; Supplementary Fig. 1d) on adult day 3 demonstrated significantly increased mitochondrial superoxide in both TDP-43 and *har-1*^−/−^ strains (Fig. 1f,g). Despite the increase in mitosox-red intensity in *har-1*^−/−^ worms on adult days 3 and 7, no significant differences in mitosox-red were seen on adult day 1 compared to N2 controls (Supplementary Fig. 1e), indicative of an aging but not developmental alteration. Given the important role of mitochondrial health in *C. elegans* longevity^23^, we performed lifespan analysis of age-matched N2 and *har1*^−/−^ strains at 16 °C. While all N2 worms died over a 33-day period after hatching, the *har-1*^−/−^ strain showed a significantly reduced lifespan (median survival 22 days) compared to N2 controls (median survival 28 days; Fig. 1h), indicating that endogenous *har-1* contributes to lifespan of *C. elegans*.

### Human CHCHD10 but not FTD/ALS mutations complement the loss of *har-1*

**Figure 2:**
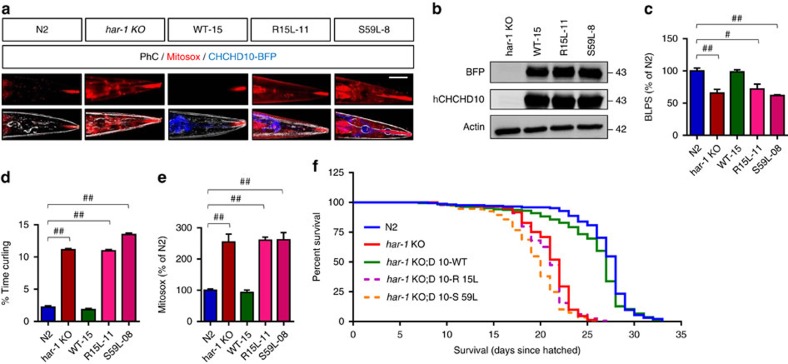
Wild-type human CHCHD10 but not FTD/ALS mutations complement the loss of har-1. (**a**) Representative images of age-synchronized worms stained with 5 μM mitosox-red and imaged for BFP (CHCHD10), mitosox-red and phase contrast (PhC) by confocal microscopy. Scale bar, 100 μm. (**b**) Representative blots of BFP and hCHCHD10 expression by immunoblotting. (**c**,**d**) Age-synchronized N2, *har-1* KO and *har-1* KO worms expressing WT, R15L or S59L CHCHD10 transferred to fresh NGM plate or M9 buffer and videotaped to measure motility (BLPS, *n*=50 worms/strain) and curling behaviour (*n*=50 worms/strain) at normalized to N2 control (one-way ANOVA, *post hoc* Tukey, ^#^*P*<0.001, ^##^*P*<0.0001). (**e**) Mitosox-red intensity (head region excluding mouth, pharynx and intestine) at 22 °C normalized to N2 control in adult day 3 worms (one-way ANOVA, *post hoc* Tukey, ^##^*P*<0.0001, *n*=29 worms/strain). (**f**) Lifespan analysis (% of worms surviving since days hatched) of N2, *har-1* KO, *har-1* KO worms expressing CHCHD10 variants at 16 °C (data from WT-2/WT-14/WT-15 combined, R15L-3/R15L-10/R15L-11 combined and S59L-2/S59L-18/S59L-08 combined) (Log-rank Mantel–Cox test, ^##^*P*<0.0001, *n*=170–190 worms/genotype). All quantitative graphs with error bars were expressed as mean±s.e.m. ANOVA, analysis of variance; KO, knockout.

To determine whether human CHCHD10 can complement phenotypes in the *har-1*^−/−^ background, we engineered *C. elegans* DNA constructs expressing human CHCHD10-blue fluorescent protein (BFP) variants under the control of the ubiquitous *eef-1A.1* promoter. These constructs were then microinjected into the gonad of *har-1*^−/−^ *C. elegans*. Upon verification of generational transmission by BFP fluorescence in their progeny (Fig. 2a), locomotion, mitosox-red, and longevity assays were carried out. We initially chose three independent *har-1*(*gk3124*) rescue lines (WT-02, WT-14 and WT-19) with moderate to high BFP fluorescence. In all three clonal strains, crawling speeds were significantly rescued by human CHCHD10 (Supplementary Fig. 2a). In liquid curling tests, abnormal curling was also rescued in all three hCHCHD10-expressing strains (Supplementary Fig. 2b). Consistent with these data, the *har-1*^−/−^(CHCHD10–02) strain, which exhibited moderate BFP fluorescence, also fully reduced mitosox-red staining to N2 control levels (Supplementary Fig. 2c). Indeed, we confirmed the expression of CHCHD10 by western blotting using antibodies against BFP and hCHCHD10 (Supplementary Fig. 2d).

We next injected *har-1*^−/−^ strains with WT and FTD/ALS-linked CHCHD10 mutations (R15L and S59L) and chose strains that contained comparable BFP fluorescence and CHCHD10/BFP expression by immunoblotting (Fig. 2a,b). WT but not R15L or S59L strains rescued the slow motility and abnormal curling phenotypes associated with *har-1*^−/−^ (Fig. 2c,d), indicating that both mutations exhibit a loss of function phenotype with regards to *har-1* complementation. While WT hCHCHD10 reduced mitochondrial superoxide (mitosox-red) down to control N2 levels, R15L and S59L mutants completely failed to rescue this phenotype (Fig. 2a,e). Similar results on crawling, curling, and mitosox-red tests were obtained from additional clonal strains (Supplementary Fig. 2d–i). Interestingly, the S59L-18 mutant showed a curling phenotype that was significantly worse than the parental *har-1*^−/−^ strain (Supplementary Fig. 2g), suggesting that the S59L mutation may also contains an additional gain-of-function component. Likewise, in liquid thrashing tests, both R15L and S59L mutants showed a modest but significant decline in thrashing rates compared the parental *har-1*^−/−^ strain (Supplementary Fig. 2h). Lifespan analysis at 16 °C showed that while three strains of WT hCHCHD10 (WT-02, WT-14 and WT-15 combined) prolonged lifespan to control N2 levels, 3 strains of R15L (R15L-03, R15L-10 and R15L-11 combined) and S59L (S59L-02, S59L-18 and S59L-08 combined) failed to rescue the reduced lifespan phenotype in the *har-1*^−/−^ background (Fig. 2f). The median survival times for N2 and *har-1*^−/−^; D10-WT strains were 27–28 days, whereas those for *har-1*^−/−^ (22 days), *har-1*^−/−^;D10-R15L (21 days), and *har-1*^−/−^; D10-S59L (20 days) were significantly shorter.

### CHCHD10 dysfunction promotes mitochondrial and synaptic deficits

**Figure 3:**
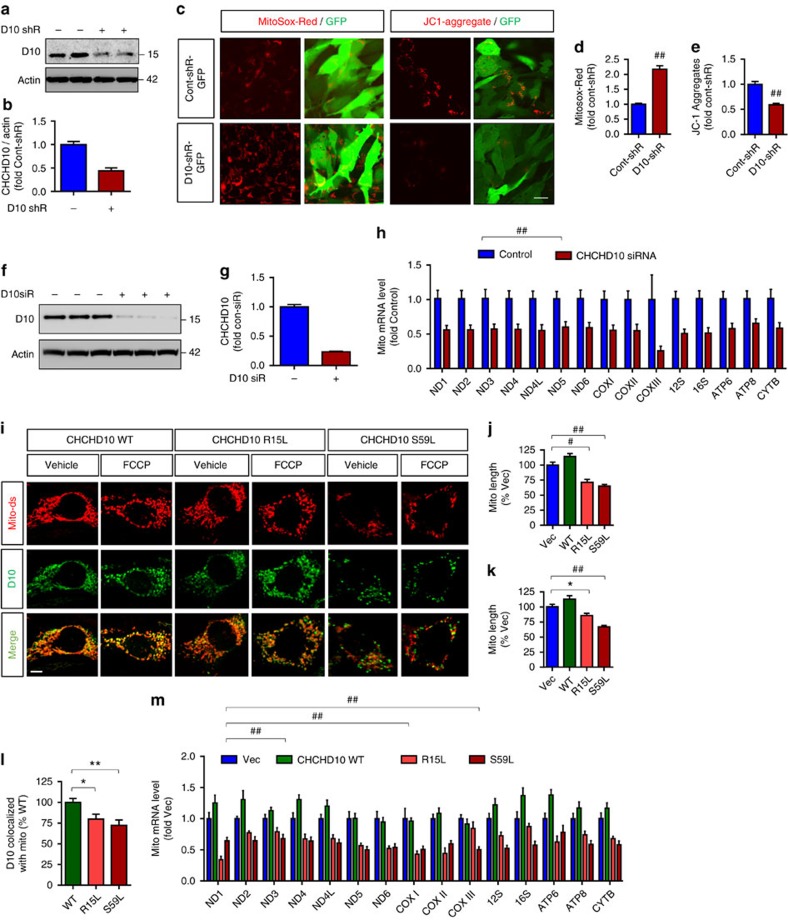
CHCHD10 depletion and FTD/ALS CHCHD10 mutations drive mitochondrial dysfunction. (**a**,**b**) Representative blots and quantification of CHCHD10 knockdown in CHCHD10 shRNA transduced HT22 cells. (**c**–**e**) Mouse hippocampus-derived HT22 neuroblastoma cells transduced with control shRNA/GFP or CHCHD10 (D10) shRNA/GFP lentivirus and stained for mitosox-red and JC-1 aggregates. (**c**) Representative images of mitosox-red and JC-1 aggregate staining in GFP expressing cells. Scale bar, 10 μm. (**d**,**e**) Quantification of average mitosox-red and JC-1 aggregate intensity per cell in GFP-positive cells (≥20 randomly chosen cells/replicate) normalized to control (*t*-test, ^##^*P*<0.0001, *n*=6 replicates). (**f**,**g**) Representative blots and quantification of CHCHD10 knockdown by CHCHD10 siRNA in HEK293T cells. (**h**) HEK293T cells transfected with control or CHCHD10 siRNA and subjected to qRT–PCR for the indicated mitochondria-encoded transcripts normalized to control (paired *t*-test for transcripts combined, ^##^*P*<0.0001, *n*=4 replicates). (**i**–**l**) HT22 cells co-transfected with mito-dsRed and Flag-CHCHD10 variants (WT, R15L and S59L), treated with/without FCCP (1 μM) for 1 h, stained for Flag (M2), and subjected to confocal imaging for mito-dsRed and CHCHD10 variants. (**i**) Representative images of mito-dsRed, and CHCHD10 (Flag M2) fluorescence. Scale bar, 5 μm. (**j**,**k**) Quantification of mitochondrial length without FCCP (**j**) and with FCCP (**k**) by Image J normalized to vector control (one-way ANOVA, posthhoc Tukey, *P<0.05, ^#^*P*<0.001, ^##^*P*<0.0001, *n*=6 replicates). (**l**) Quantification of Flag-CHCHD10 co-localized with mito-dsRed by Image J normalized to WT control (one-way ANOVA, *post hoc* Tukey, *P<0.05, **P<0.01, *n*=6 replicates). (**m**) 293T cells transfected with control or vector control or Flag-CHCHD10 variants (WT, R15L and S59L) and subjected to qRT–PCR for the indicated mitochondria-encoded transcripts normalized to vector control (two-way ANOVA for transcripts combined, *post hoc* Tukey, ^##^*P*<0.0001; Tukey multiple comparison for individual genes at **P<0.01 (WT versus R15L: all genes except COXIII; WT versus S59L: all genes; *n*=6 replicates)). All quantitative graphs with error bars were expressed as mean±s.e.m. ANOVA, analysis of variance.

Bannwarth *et al*.^13^ reported that the CHCHD10 S59L mutation reduces mitochondrial length and promotes disorganization of mitochondrial cristae structure. However, the effects of endogenous CHCHD10 depletion have not been investigated in mammalian cells. Thus, we utilized lentiviruses expressing mouse CHCHD10 shRNA together with green fluorescent protein (GFP) or GFP alone to knockdown endogenous CHCHD10 in mouse HT22 cells, a hippocampus-derived neuroblastoma cell line that has been characterized for mitochondrial dysfunction and cell death^24^,^25^. Forty-eight hours after transduction, immunoblotting indeed confirmed that CHCHD10 shRNA lentivirus transduced cells contained markedly less endogenous CHCHD10 by >60% overall (Fig. 3a,b). Next, we measured mitochondrial superoxide (mitosox-red) and membrane potential (ΔΨm, JC-1 aggregates) in GFP expressing HT22 cells. JC-1 aggregates indicate healthy polarized mitochondria (red), whereas JC-1 monomers (green) indicate unhealthy depolarized mitochondria^26^. Although we were unable to measure JC-1 monomers due to its spectral overlap with GFP fluorescence, depletion of endogenous CHCHD10 by shRNA in GFP+ cells significantly increased mitosox-red intensity by >2-fold (Fig. 3c,d) and reduced JC-1 aggregates by ∼50% (Fig. 3c–e). This phenotype was not attributable to the related gene CHCHD2, as knockdown of CHCHD10 did not change CHCHD2 mRNA as measured by quantitative PCR reverse-transcription (qRT–PCR) (Supplementary Fig. 3a,b). Real-time qRT–PCR analysis of 15 genes encoded in mitochondria also demonstrated that knockdown of endogenous CHCHD10 (Fig. 3f,g) reduced levels of all 15 transcripts by 40–75% and significantly reduced mitochondrial transcripts overall (Fig. 3h), indicating a general perturbation in mitochondrial health.

To visualize the localization of CHCHD10 and assess mitochondrial integrity secondary to WT or FTD/ALS mutant CHCHD10 expression, we next co-transfected NIH3T3 cells with the mitochondrial marker mito-dsRed and wild-type (WT) or FTD/ALS-linked (R15L or S59L) CHCHD10 variants. We chose the NIH3T3 cells for this purpose due to their larger size and flatter shape than HT22 cells to better visualize mitochondrial morphology. As anticipated, all CHCHD10 variants were largely co-localized with mito-dsRed (Fig. 3i). Whereas WT CHCHD10 showed visibly elongated mitochondria, R15L and S59L CHCHD10 mutants displayed significantly fragmented and shortened mitochondria (Fig. 3i,j), which often did not co-localize with mito-dsRed. Quantification showed that the R15L and S59L mutants co-localized significantly less with mito-dsRed compared to its WT counterpart (Fig. 3i–l). Upon treatment with the mitochondrial uncoupling agent FCCP (1 μM) for 1 h, fragmentation of mitochondria were seen in all conditions, although mitochondrial lengths were still shorter in R15L and S59L mutants compared to vector or WT CHCHD controls (Fig. 3i,k). WT CHCHD10 transfected cells showed a nonsignificant trend toward increased mitochondrial length compared to vector control transfected cells both at basal and carbonyl cyanide 4-(trifluoromethoxy)phenylhydrazone (FCCP) conditions (Fig. 3i–k). Real-time qRT–PCR analysis demonstrated that like CHCHD10 knockdown, R15L and S59L mutations significantly reduced mitochondrial transcripts overall compared to vector or WT controls (Fig. 3m). Fourteen out of 15 individual mitochondrial encoded transcripts were significantly reduced by the R15L mutation, and all 15 transcripts were significantly reduced by the S59L mutation compared to WT CHCHD10 (Fig. 3m). In contrast, WT CHCHD10 significantly increased mitochondrial transcripts overall compared to vector control (Fig. 3m) and showed a nonsignificant trend for increasing 11/15 transcripts individually compared to vector control (Fig. 3m), indicating that expression of WT CHCHD10 can augment endogenous CHCHD10 function, whereas R15L and S59L mutations display a phenotype like that of CHCHD10 depletion.

**Figure 4:**
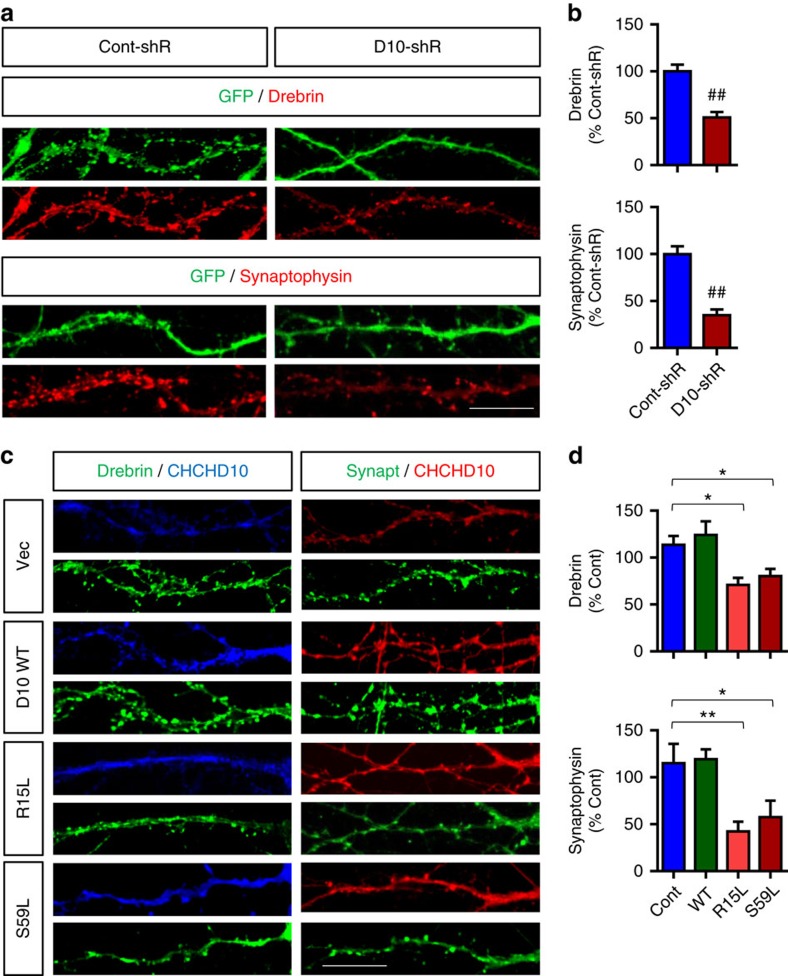
CHCHD10 depletion and FTD/ALS CHCHD10 mutations compromise synaptic integrity in primary neurons. (**a**,**c**) Hippocampal primary neurons transduced with control shRNA/GFP lentivirus, CHCHD10 shRNA/GFP lentivirus or CHCHD10 rAAV9 variants (WT, R15L and S59L) and subjected to staining for drebrin, synaptophysin and/or CHCHD10 followed by confocal imaging. Representative images of primary neurites shown. Scale bar, 20 μm. (**b**) Quantification of average overall neuritic drebrin and synaptophysin signals minus average diffuse neuritic staining (non-puncta) normalized to control in GFP^+^ neurons (*t*-test, ^##^*P*<0.0001, *n*=6 replicates, ≥30 randomly chosen neurites/replicate). (**d**) Quantification of average overall neuritic drebrin and synaptophysin signals minus average diffuse neuritic staining (non-puncta) and normalized to control (one-way ANOVA, *post hoc* Tukey, *P<0.05, **P<0.01, *n*=6 replicates, ≥30 randomly chosen neurites/replicate). All quantitative graphs with error bars were expressed as mean±s.e.m. ANOVA, analysis of variance.

Neuronal synapses are energy-intensive compartments highly enriched in mitochondria, which are transported in an activity-dependent manner^27^. To determine whether the mitochondrial dysfunction associated with CHCHD10 can been seen at the level of synaptic integrity, we transduced DIV15 hippocampal primary neurons with control GFP or CHCHD10-shRNA/GFP lentiviruses. Compared to GFP only transduced neurons, CHCHD10-shRNA/GFP-transduced neurons showed significantly reduced intensity of drebrin (postsynaptic marker) and synaptophysin (presynaptic marker) puncta in neuronal processes on DIV21 (Fig. 4a,b). We also transduced primary neurons with rAAV9 expressing empty vector or CHCHD10 variants (WT, R15L and S59L). R15L or S59L transduced neurons showed significantly reduced drebrin and synaptophysin puncta compared to empty vector or WT CHCHD10 transduced neurons on DIV21 (Fig. 4c,d), indicating that these FTD/ALS-linked CHCHD10 mutants display a synaptic phenotype similar to that seen with CHCHD10 depletion.

### TDP-43 interacts with CHCHD10 and promotes its nuclear localization

**Figure 5:**
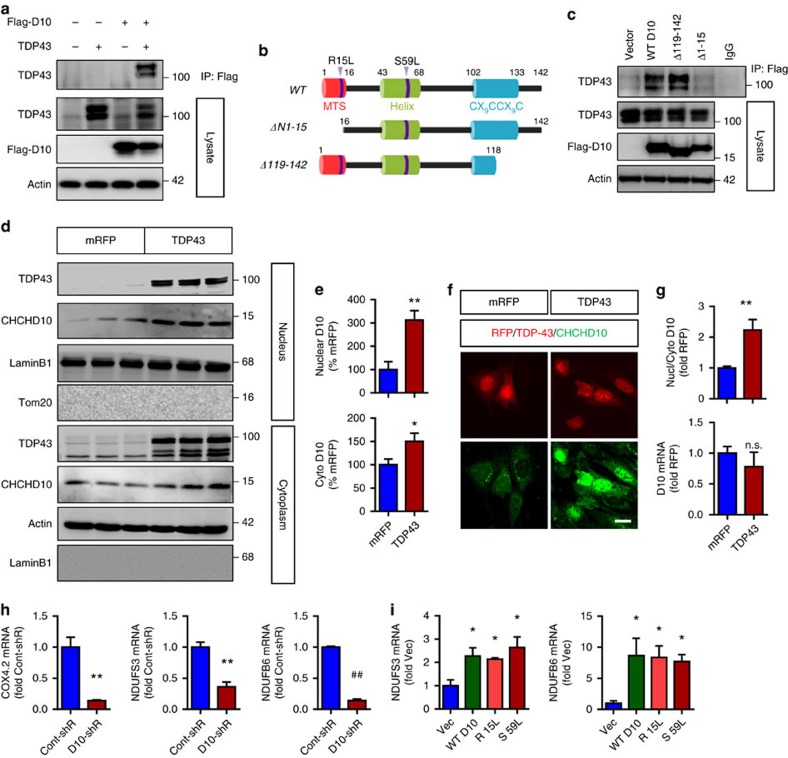
TDP-43 forms a complex with CHCHD10 and promotes its nuclear localization. (**a**,**c**) HT22 cells transfected with/without TDP-43-tomato and the indicated Flag-CHCHD10 variants, and lysates subjected to co-IP with Flag (M2) antibody and/or immunoblotting for the indicated proteins. Note that the loss of N1-15 residues abrogates CHCHD10 pull down with TDP-43. (**b**) Schematic representation of CHCHD10 domains, mutations and deletions. (**d**,**e**) HEK293T cells transfected with mRFP or TDP-43-tomato, biochemically fractionated for nucleus versus cytoplasm, and subjected to immunoblotting for the indicated proteins. Numbers right of the blots indicate kDa markers. Note that the antibody against TDP-43 recognizes N-terminal residues 1–89, indicating that slightly faster migrating TDP-43-tomato fragment(s) likely represent cleavages(s) within the tomato fluorescent protein. (**e**) Quantification of nuclear and cytoplasmic TDP-43 normalized to mRFP control (*t*-test, *P<0.05, **P<0.01, *n*=6 replicates) (**f**,**g**) HT22 cells transfected with mRFP or TDP-43-tomato and subjected to ICC for endogenous CHCHD10 and direct fluorescence for mRFP or TDP-43-tomato. Scale bar, 10 μm. (**g**) Quantification of nuclear/cytoplasmic ratio normalized to control (top graph; *t*-test, **P<0.01, *n*=6 replicates) and qRT–PCR for endogenous CHCHD10 transcript (bottom graph, *n*=4 replicates). (**h**) HT22 cells transduced with control shRNA/GFP or CHCHD10 shRNA/GFP lentivirus and subjected to qRT–PCR for the indicated nuclear transcripts (COX4.2, NDUFS3 and NDUFB6) and normalized to control (*t*-test, **P<0.01, ^##^*P*<0.0001, *n*=4 replicates). (**i**) HT22 cells transfected with vector control or CHCHD10 variants (WT, R15L and S59L) and subjected to qRT–PCR for the indicated nuclear transcripts (NDUFS3 and NDUFB6) and normalized to control (one-way ANOVA, *post hoc* Tukey, *P<0.05 compared to Vec, *n*=4 replicates). All quantitative graphs with error bars were expressed as mean±s.e.m. ANOVA, analysis of variance.

TDP-43 is normally a nuclear protein that can translocate to the cytoplasm and mitochondria under pathological conditions^3^,^4^. Thus, we first assessed whether endogenous CHCHD10 can form a physical complex with endogenous TDP-43. Whereas IgG beads alone failed to pull down either endogenous CHCHD10 or TDP-43, CHCHD10 immune complexes contained endogenous TDP-43, and TDP-43 immune complexes also contained endogenous CHCHD10 (Supplementary Fig. 4a). In HT22 cells transfected with vector, Flag-CHCHD10 and/or TDP-43-tomato, M2-Flag immune complexes contained TDP-43-tomato, whereas no TDP-43-tomato was present in M2-Flag immune complexes from TDP-43-tomato only transfected cells (Fig. 5a). We next generated CHCHD10 constructs lacking N-terminal 16 residues (Δ1–15) or lacking C-terminal residues 119–142 (Δ119–142). Residues 1–15 contain a hypothetical mitochondrial targeting sequence^28^, whereas residues 119–142 contain the CX_9_CCX_9_C structural motif (Fig. 5b). Co-transfection of CHCHD10 variants with vector control or TDP-43-tomato demonstrated that while WT and Δ119–142 variants similarly formed complexes with TDP-43, the Δ1–15 variant failed to interact with TDP-43 (Fig. 5c). Like WT CHCHD10, mutant CHCHD10 R15 and S59L formed complexes with TDP-43 (Supplementary Fig. 4b). Given the physical association of TDP-43 with CHCHD10 and the nuclear localization of TDP-43, we tested whether TDP-43 overexpression alters endogenous CHCHD10 localization. Surprisingly, TDP-43-tomato transfection increased nuclear localization of CHCHD10 by a significant three-fold while having a marginal effect on cytoplasmic CHCHD10 in biochemical fractionation experiments (Fig. 5d,e). The mitochondrial protein TOM20 and nuclear protein LaminB1 were not detected in nuclear and cytoplasmic fractions, respectively (Fig. 5d). In accord with biochemical observations, the ratio of endogenous nuclear versus cytoplasmic CHCHD10 was significantly increased by ∼2.3-fold in TDP-43-tomato transfected cells as measured by immunofluorescence (Fig. 5f,g), while having no effect on endogenous CHCHD10 transcript (Fig. 5g, bottom graph).

CHCHD2, a protein with high homology to CHCHD10 and recently shown to be mutated in Parkinson's and Lewy body diseases^29^,^30^,^31^, can shuttle between mitochondria and nucleus and function as a nuclear co-transactivator under conditions of hypoxia^32^,^33^. Thus, we tested whether the sets of nuclear genes altered by CHCHD2 are also similarly altered by CHCHD10 knockdown or overexpression. Indeed, cytochrome c oxidase subunit-4 isoform-2 (COX4.2), NADH dehydrogenase iron-sulfur protein 3 (NDUFS3), and NADH dehydrogenase-1β sub-complex subunit 6 (NDUFB6) transcripts (nuclear-encoded mitochondrial proteins) were all significantly reduced by CHCHD10 shRNA transduction (Fig. 5h), similar to that seen with CHCHD2 knockdown^33^. However, this was not attributable to CHCHD2 expression, as CHCHD10 knockdown had no effect on CHCHD2 transcript levels (Supplementary Fig.3b). Conversely, CHCHD10 overexpression significantly increased NDUFS3 and NDUFB6 transcripts, but such increases did not significantly differ among WT, R15 and S59L variants (Fig. 5i), suggesting that the putative nuclear transactivation function of CHCHD10 may not be profoundly altered by the presence of R15L and S59L mutations. Nonetheless, these novel data indicate that TDP-43 can form physical complexes with CHCHD10, alter its subcellular localization, and potentially regulate CHCHD10-mediated nuclear function.

### CHCHD10 dysfunction induces cytoplasmic TDP-43 accumulation

**Figure 6:**
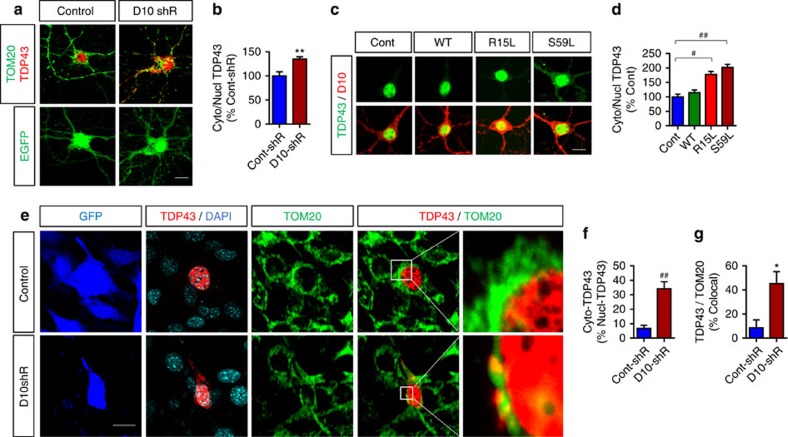
CHCHD10 depletion and FTD/ALS CHCHD10 mutations induce cytoplasmic TDP-43 mislocalization. (**a**,**b**) Primary hippocampal neurons transduced with control shRNA/GFP or CHCHD10/GFP shRNA lentivirus and subjected to ICC for endogenous TDP-43 or direct GFP fluorescence (TOM20 originally blue pseudocolored to green; **a**, upper panels). Scale bar, 10 μm. (**b**) Quantification of cytoplasmic versus nuclear TDP-43 ratio normalized to control (*t*-test, **P<0.01, *n*=6 replicates). (**c**,**d**) Primary hippocampal neurons transduced with control rAAV9 or CHCHD10 rAAV9 variants and subjected to ICC for endogenous TDP-43, direct GFP fluorescence, and/or CHCHD10. Scale bar, 10 μm. (**d**) Quantification of cytoplasmic versus nuclear TDP-43 ratio normalized to control (one-way ANOVA, *post hoc* Tukey, ^#^*P*<0.001, ^##^*P*<0.0001, *n*=6 replicates). (**e**–**g**) NIH3T3 cells transduced with control shRNA/GFP or CHCHD10/GFP shRNA lentivirus and transfected with TDP-43-tomato followed by ICC for TOM20 and direct fluorescence for GFP, TDP-43-tomato, and DAPI. White outlined boxes magnified in right panels (TOM20 originally blue pseudo-coloured to green and GFP pseudo-coloured to blue). Scale bar, 10 μm. (**f**,**g**) Quantification of cytoplasmic TDP-43 as a percentage of nuclear TDP-43 and co-localization of cytoplasmic TDP-43 with TOM20 by Image J (*t*-test, *P<0.05, ^##^*P*<0.0001, *n*=6 replicates). ANOVA, analysis of variance.

To gain insights into the normal activity of CHCHD10 on TDP-43 localization, we transduced primary mouse hippocampal neurons with control GFP or CHCHD10 shRNA/GFP lentiviruses and subjected neurons for immunocytochemistry (ICC) for TOM20 (mitochondria marker—pseudo-coloured to green) and TDP-43 (red). In GFP only transduced neurons, endogenous TDP-43 was exclusively localized to the nucleus (Fig. 6a, left upper panel). However, in neurons transduced with CHCHD10 shRNA/GFP, endogenous TDP-43 was not only localized to the nucleus but also in the cytoplasm and neuritic processes (Fig. 6a, right upper panel). Quantification of nuclear and cytoplasmic TDP-43 staining demonstrated a significant >30% increase in the ratio of cytoplasmic/nuclear TDP-43 in CHCHD10 shRNA/GFP compared to GFP-only transduced neurons (Fig. 6b). We also transduced primary neurons with rAAV9 expressing empty vector control, WT, R15L or S59L CHCHD10 variants. Compared to control and WT CHCHD10, neurons transduced with R15L or S59L CHCHD10 mutants also demonstrated significant increases in the ratios of endogenous cytoplasmic/nuclear TDP-43 staining (Fig. 6c,d).

To assess TDP-43 localization in a different cellular system with exogenous TDP-43 expression, NIH3T3 cells were transduced with control shRNA/GFP or CHCHD10 shRNA/GFP lentivirus and then transfected with TDP-43-tomato. Much like that seen in neurons, TDP-43-tomato was nearly exclusively nuclear in control shRNA/GFP-transduced cells (Fig. 6e). However, CHCHD10 shRNA/GFP-transduced cells exhibited significantly increased ratio of cytoplasmic/nuclear tomato-TDP-43 fluorescence (Fig. 6e,f). The cytoplasmic TDP-43-tomato exhibited punctate and aggregated appearance, which often (∼45%) but not exclusively co-localized with the mitochondrial marker TOM20 (Fig. 6e,g). Biochemical solubility separation experiments showed that CHCHD10 knockdown significantly increased TDP-43-tomato in the SDS-insoluble but not in the SDS-soluble fraction (Supplementary Fig. 5a,b).

**Figure 7:**
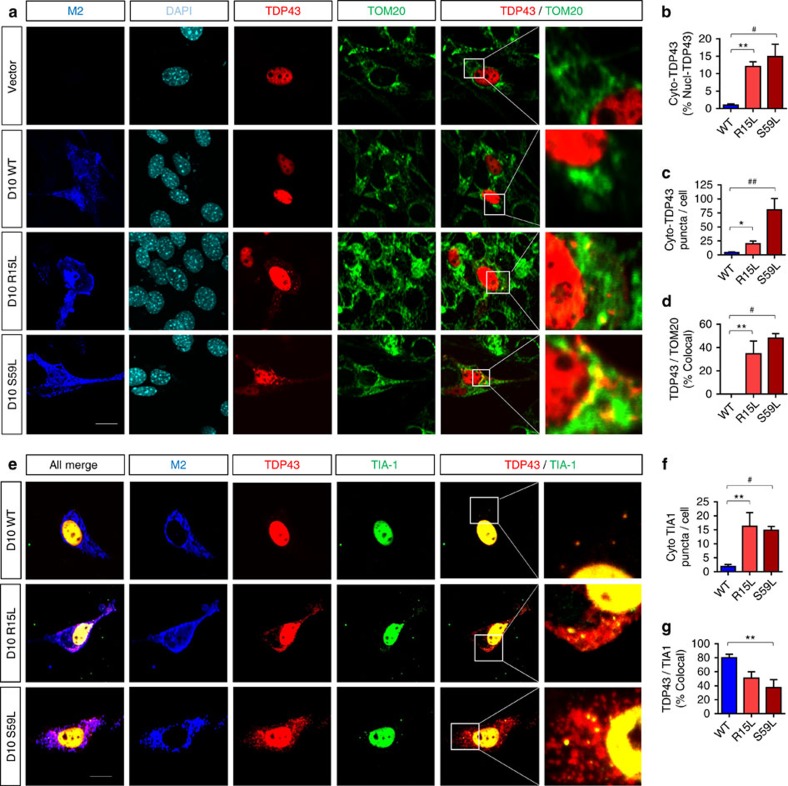
FTD/ALS CHCHD10 mutations induce TDP-43 mislocalization frequently with mitochondria. (**a**–**g**) NIH3T3 cells co-transfected with TDP43-tomato and Flag-CHCHD10 variants (WT, R15L and S59L) and subjected to ICC for Flag (M2), Tia-1, and/or TOM20 and direct fluorescence for TDP-43-tomato and DAPI. White outlined boxes magnified at far right panels. Scale bar, 10 μm. (**b**–**d**,**f**,**g**) Quantification of cytoplasmic TDP-43-tomato as a percentage of nuclear TDP-43-tomato, cytoplasmic TDP-43-tomato puncta per cell, co-localization of TDP-43 with TOM20 or Tia-1, and cytoplasmic Tia-1 puncta per cell (1-way ANOVA, *post hoc* Tukey, *P<0.05, **P<0.01, ^#^*P*<0.001, ^##^*P*<0.0001, *n*=6 replicates). All quantitative graphs with error bars were expressed as mean±s.e.m. ANOVA, analysis of varaince.

We next co-transfected NIH3T3 cells with TDP-43-tomato and Flag-CHCHD10 variants (WT, R15L and S59L) and examined TDP-43-tomato localization with mitochondria (TOM20) and Tia-1+ granules. Cytoplasmic Tia-1 granules, so called ‘stress granules', represent stress-induced punctate structures that sequester stalled mRNAs and various proteins, which is generally thought to be part of an initially protective response^34^. Expression of WT CHCHD10 demonstrated nearly exclusive localization of TDP-43-tomato to the nucleus (Fig. 7a). However, transfection of R15L and S59L mutations resulted in significant mislocalization of TDP-43-tomato to the cytoplasm (Fig. 7a–d). Quantification of overall TDP-43-tomato intensity in the cytoplasm versus nucleus and numbers of TDP-43-tomato puncta/cell demonstrated significant increases by R15L and S59L mutations as compared to WT control (Fig. 7b–d). These often irregularly shaped cytoplasmic TDP-43-tomato cytoplasmic puncta seen in R15L and S59L expressing cells were 35–45% co-localized with TOM20 (Fig. 7a–d). While Tia-1+ puncta per cell were dramatically increased by R15L and S59L mutations compared to WT control (Fig. 7e,f), these mutations tended to decrease the co-localization of TDP-43-tomato with Tia-1+ granules (Fig. 7g). While either none or very few TDP-43-tomato and Tia-1 puncta were seen in WT CHCHD10 transfected cells, those cytoplasmic TDP-43-tomato puncta present were largely co-localized with Tia-1+ puncta (Fig. 7g). In all cases, Tia-1+ puncta were tiny in size compared to the irregularly shaped cytoplasmic TDP-43-tomato immunoreactivity, which often co-localized with mitochondria. Intriguingly, the loss of either N-terminal 1–15 or C-terminal 119–142 residues of CHCHD10 S59L significantly reduced both the ratio of cytoplasmic/nuclear TDP-43-tomato and TDP-43-tomato puncta per cell compared to full-length S59L, indicating that the S59L mutation functions in concert with these domains to exhibit its pathogenic activity (Supplementary Fig. 6a,b). However, the loss of these N- and C-terminal domains did not significantly reduce cytoplasmic Tia-1+ puncta per cell (Supplementary Fig. 6a–c). While N-terminal 16 residues of CHCHD10 have been hypothesized to comprise a mitochondrial targeting sequence^28^, deletion of either the C-terminal or N-terminal domain significantly compromised CHCHD10 co-localization with mitochondria, although the effect of the C-terminal deletion was more pronounced (Supplementary Fig. 7a,b)

### CHCHD10 variants alter TDP-43-induced apoptosis and synaptic damage

**Figure 8:**
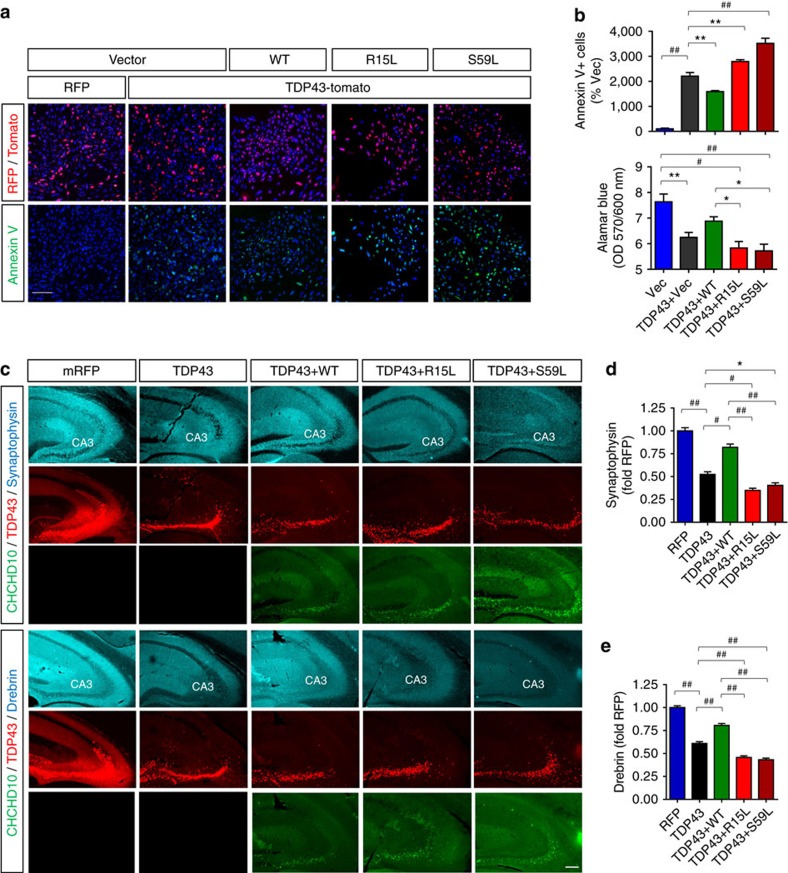
Wild-type CHCHD10 ameliorates and FTD/ALS CHCHD10 mutations potentiate TDP-43-induced apoptosis and synaptic impairment. (**a**,**b**) HT22 neuroblastoma cells transfected with TDP-43-tomato and/or Flag-CHCHD10 variants, then subjected to Annexin V staining and direct TDP-43-tomato fluorescence. Cells also subjected to alamarBlue staining and quantification of OD570/600 ratio. Scale bar, 100 μm. (**b**) Quantification of Annexin V+ cells in RFP+ or TDP-43-tomato+ cells normalized to control (upper graph: one-way ANOVA, *post hoc* Tukey, **P<0.01, ^##^*P*<0.0001, *n*=6 replicates). Quantification of alamarBlue OD570/600 ratio by microplate reader (lower graph: one-way ANOVA, *post hoc* Tukey, *P<0.05, **P<0.01, ^#^*P*<0.001, ^##^*P*<0.0001, *n*=6 replicates). (**c**–**e**) C57BL6 mice (2mo) transduced with purified high-titer TDP-43-tomato rAAV9 and/or GFP-CHCHD10 rAAV9 variants by stereotaxic injection into the hippocampus. Brains processed for direct confocal microscopy for TDP-43-tomato and GFP-CHCHD10 as well as indirect IHC for synaptophysin (presynaptic) and drebrin (postsynaptic) 1 month post injection. Scale bar, 100 μm. (**d**,**e**) Quantification of synaptophysin and drebrin intensity performed from the stratum lucidum (SL) of CA3 (one-way ANOVA, *post hoc* Tukey, *P<0.05, **P<0.01, ^#^*P*<0.001, ^##^*P*<0.0001, *n*=4,5 mice/grp). All quantitative graphs with error bars were expressed as mean±s.e.m. ANOVA, analysis of variance.

To determine the effects of CHCHD10 variants on TDP-43-induced cell death, we transfected HT22 cells with/without TDP-43-tomato and Flag-CHCHD10 variants (WT, R15L or S59L) and subjected cells to Annexin V staining and alamarBlue assay, an early marker of apoptosis and a general marker of cell viability, respectively. As expected, TDP-43-tomato transfected cells exhibited a dramatic (>2,000%) increase in Annexin V+ cells and a significant reduction in alamarBlue OD570/600 nm ratio (Fig. 8a,b). However, co-transfection of TDP-43-tomato with WT CHCHD10 significantly ameliorated TDP-43-induced increase in Annexin V+ cells and decrease in alamarBlue cell viability index, whereas both R15L and S59L mutants significantly exacerbated TDP-43-induced cell death compared to WT CHCHD10 (Fig. 8a,b). To explore this phenotype *in vivo* in regards to synaptic integrity, we next generated and purified high-titer (>1 × 10^12^ vg ml^−1^) rAAV9 expressing mRFP control, TDP-43-tomato, or GFP-CHCHD10 variants (WT, R15L or S59L). Combinations of these purified rAAV9 were then stereotaxically injected bilaterally into the hippocampus of 2-month-old mice. One-month post injection, we processed the brains for detection of GFP-CHCHD10, TDP-43-tomato and synaptic integrity. Synaptophysin was chosen as a presynaptic marker, whereas drebrin was chosen as a postsynaptic marker. Both TDP-43-tomato and GFP-CHCHD10 variants were expressed largely within the dentate gyrus and the CA3 layer of the hippocampus (Fig. 8c). Quantification of synaptophsyin and drebrin intensity within the stratum lucidum (synaptic layer) of CA3 demonstrated that TDP-43-tomato expression dramatically reduced synaptophysin and drebrin immunoreactivity (IR) by 50% and 39%, respectively (Fig. 8c–e). However, co-expression of TDP-43-tomato with WT CHCHD10 significantly prevented the decline in synaptophysin and drebrin IR (Fig. 8c–e). In contrast, co-expression of TDP-43-tomato with R15L or S59L mutations significantly exacerbated the decrease in synaptophysin and drebrin IR induced by TDP-43-tomato (Fig. 8c–e). These results taken together indicate that WT CHCHD10 blocks synaptic toxicity associated with TDP-43, whereas FTD/ALS mutants (R15L and S59L) worsen TDP-43-induced synaptic pathology.

## Discussion

Recent studies have shown the linkage and/or association of multiple *CHCHD10* mutations with the spectrum of familial and sporadic FTD–ALS diseases^13^,^14^,^15^,^16^,^17^ as well as the related Charcot–Marie–Tooth disease type 2 (ref. 18) and spinal muscular atrophy Jokela type^19^ disorders. Despite previous observations that *CHCHD10* encodes a protein largely localized to the intermembrane space of mitochondria in the MICOS complex^14^ and evidence that the S59L mutation reduces mitochondrial length and disorganizes mitochondrial cristae morphology^13^, neither the normal function of endogenous CHCHD10 nor the nature of the FTD/ALS-associated CHCHD10 mutations have been investigated. Furthermore, whether and how CHCHD10 alters the pathological milieu (that is, TDP-43 pathology) of FTD/ALS spectrum diseases have not been scrutinized. In this study, we made a series of novel observations, utilizing *C. elegans*, cell lines, mouse primary neurons and mouse brains, implicating the normal protective role of CHCHD10 in mitochondrial and synaptic integrity, whereas FTD/ALS-associated mutations exhibit loss of function and/or dominant negative activities. Moreover, our results have for the first time tied together the activities of CHCHD10 to cytoplasmic TDP-43 inclusions, which are the principle pathological lesions in the vast majority of ALS and FTLD-TDP. Specifically, we found that *C. elegans* deficient in *har-1*, the closest orthologue to human *CHCHD10*, display severe abnormalities in locomotion and reduced lifespan associated with mitochondrial superoxide overproduction, which were completely rescued by WT CHCHD10 but not FTD/ALS-associated CHCHD10 mutations (R15L and S59L). In mammalian cells, overexpression of ALS/FTD-associated CHCHD10 mutations (R15L and S59L) but not WT CHDHD10 exhibited multiple abnormal mitochondrial phenotypes, nearly identical to those observed with knockdown of endogenous CHCHD10. Likewise, in primary neurons, CHCHD10 mutations (R15L and S59L) but not WT CHCHD10 reduced pre- and postsynaptic integrity similar to that seen by CHCHD10 knockdown. CHCHD10 formed physical complexes with TDP-43, an interaction that required intact N-terminal 16 residues, and TDP-43 promoted the nuclear localization of CHCHD10 in a retrograde signalling manner, which was associated with increased nuclear-encoded and mitochondrial targeted transcripts. Conversely, whereas WT CHCHD10 fully retained TDP-43 localization to the nucleus, FTD/ALS-associated CHCHD10 mutations (R15L and S59L) induced cytoplasmic mislocalization of TDP-43 in irregularly shaped inclusions often co-localized with mitochondria. Despite the significant induction of Tia-1+ granules by CHCHD10 mutations, large cytoplasmic TDP-43 inclusions were largely devoid of Tia-1, and the ability of CHCHD10 variants (mutations and deletions) to induce cytoplasmic TDP-43 and/or Tia-1 cytoplasmic inclusions were at least partially distinct. Finally, WT CHCHD10 antagonized TDP-43-induced apoptosis *in vitro* and synaptic damage *in vivo*, whereas FTD/ALS-associated CHCHD10 mutations (R15L and S59L) exacerbated such TDP-43-induced phenotypes. Our novel findings taken together therefore implicate the normal protective role of CHCHD10 in mitochondrial/synaptic integrity and inhibition of cytoplasmic TDP-43 accumulation, whereas the loss of CHCHD10 or FTD/ALS-associated CHCHD10 mutations (R15L and S59L) exhibit loss of function or dominant negative activities in these phenotypes.

The *har-1* gene in *C. elegans* represents the only orthologue to mammalian *CHCHD10* and *CHCHD2*, with nearly equal amino acid homology to both (http://useast.ensembl.org/index.html). *Har-1* was originally identified from a *C. elegans* screen to detect mutants that survive in the presence of the antimitotic drug hemiasterlin, in which the *har-1* G73E mutant was identified^22^. However, unlike the *har-1* G73E mutant that exhibits a normal lifespan, *har-1*^−/−^ *C. elegans* observed in this study were short-lived. This observation is particularly intriguing in light of the evidence that severe mitochondrial dysfunction promotes the aging process, whereas partial mitochondrial dysfunction mutants either have no effect or can activate anti-aging pathways^22^,^23^. Thus, the complete loss of *har-1* appears to be more detrimental to mitochondrial health than the *har-1* G73E mutation. Moreover, whereas no abnormal locomotion phenotype was noted in the *har-1* G73E mutant^22^, the *har-1*^−/−^ strain exhibited significant locomotion defects. Our observation that human CHCHD10 expression in the *har-1*^−/−^ background completely rescued the locomotion and mitochondrial superoxide phenotypes indicates that the core functions of *har-1* and *CHCHD10* are evolutionarily conserved. However, as we used the ubiquitous *eef-1A.1* promoter to drive CHCHD10 expression, it remains possible that the rescue phenotypes may not be identical to the use of the endogenous *har-1* promoter. Nonetheless, the same expression level of FTD/ALS mutations (R15L and S59L) was insufficient to rescue the *har-1* phenotypes, rendering them as loss of function mutations in *C. elegans*. This notion was supported by our observations in mammalian cells, primary neurons, and/or mouse brains that knockdown of CHCHD10 or expression of FTD/ALS CHCHD10 mutations produced nearly identical phenotypes with regards to mitochondrial and synaptic integrity. In general, the S59L mutation was more severe than the R15L mutation in most of the assays studied. Given that the S59L mutation was found in patients with both FTD and ALS symptoms^13^, whereas the R15L mutation was identified in patients with only ALS symptoms and some level of incomplete penetrance^16^,^35^, it is tempting to speculate that such differences may reflect the severity of phenotypes seen in this study.

**Figure 9:**
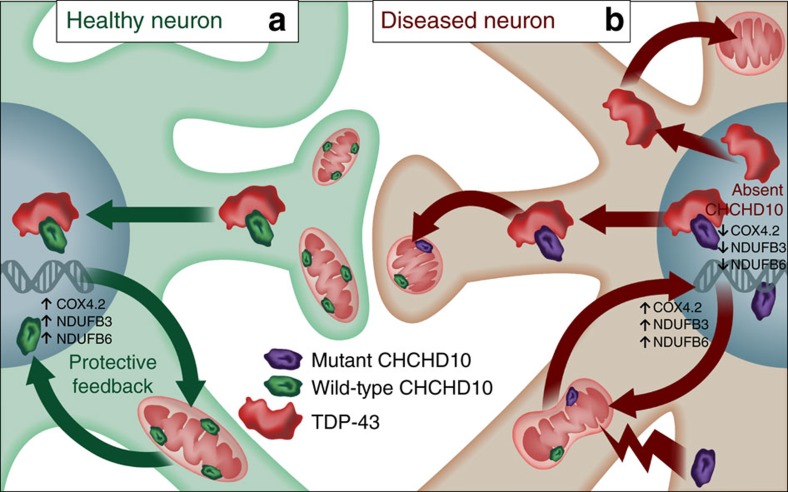
Schematic model of wild-type versus absent or mutant CHCHD10 in neurons. (**a**) In healthy neurons (green, left), CHCHD10 is normally present in the intermembrane space of mitochondria, which functions to maintain mitochondrial morphology and synaptic integrity. Upon mitochondrial stress (that is, oxidative or hypoxic), CHCHD10 retrogradely signals to the nucleus, a process facilitated by its binding to TDP-43. Nuclear CHCHD10 then promotes the transcription of mitochondrial targeted nuclear genes (that is, COX4.2, NDUFB3 and NDUFB6), which facilitates rebalancing of mitochondrial function and synaptic integrity. Meanwhile, the binding of wild-type CHCHD10 to TDP-43 prevents the exit of TDP-43 from the nucleus to cytoplasm. (**b**) In diseased neurons (brown, right), the depletion of CHCHD10 or expression of FTD/ALS mutant CHCHD10 fails to properly maintain mitochondrial morphology, thereby promoting mitochondrial fission and impairing synaptic integrity. The increase in mitochondrial stress also constitutively reduces mitochondrial CHCHD10. While mutant CHCHD10 is capable of binding to TDP-43, it is unable to effectively block the nuclear exit of TDP-43 from the nucleus to cytoplasm (similar to the absence of CHCHD10). Meanwhile, the absence of CHCHD10 reduces the transcription of mitochondrial targeted nuclear genes, whereas the increased transcription of mitochondrial targeted nuclear genes by mutant CHCHD10 is insufficient to overcome the ongoing damage incurred in mitochondria and by cytoplasmic accumulation of TDP-43, thereby compromising synaptic integrity. In the heterozygous mutant state, FTD/ALS mutations likely function primarily in a dominant negative manner, which compromises the function of endogenous CHCHD10.

The observation that multiple CHCHD2 mutations have recently been identified in Parkinson's^29^,^30^,^36^ and Lewy body disease^31^ is intriguing given that CHCHD2 is a mitochondrial protein with high similarity to CHCHD10. Like CHCHD2, which can translocate from mitochondria to the nucleus to transactivate downstream mitochondrial targeted genes^32^,^33^, CHCHD10 also positively regulated COX4.2, NDUFS3 and NDUFB6 transcripts. Although this activity on NDUFS3 and NDUFB6 transcripts did not significantly differ among WT, R15L and S59L CHCHD10 variants, we cannot rule out the possibility that other CHCHD10-regulated transcripts might be differentially altered by these or other disease-associated mutations. Nonetheless, our findings indicate that mitochondrial stress induced by the R15L or S59L mutation is likely sufficient to render neurons more vulnerable to mitochondrial and synaptic damage (Fig. 9). Although the vast majority of WT, R15L and S59L variants localized to mitochondria at steady-state, we did note a significant reduction in mitochondrial localization of R15L and S59L mutants compared to WT CHCHD10. This suggests that mitochondrial stress *per se* may reduce the targeting of CHCHD10 to mitochondria, after which CHCHD10 can undergo nuclear translocation to induce ‘protective' nuclear transcription feedback (Fig. 9).

Accumulation of cytoplasmic TDP-43 is a key pathological feature of FTLD-TDP and ALS^3^,^4^. Previous studies have identified the stable TDP-43 interactome to consist largely of RNA splicing factors, components of the transcriptional/translational machinery^37^,^38^, as well as many mitochondrial proteins^37^, the latter which are involved in mitochondrial transcription/translation, electron transport and chaperone activities. Although CHCHD10 was not identified as one of these interacting proteins from mass spectrometry studies, we found that endogenous CHCHD10 does associate with endogenous TDP-43 complexes. Moreover, our findings for the first time corroborated a molecular link between FTD/ALS-associated CHCHD10 mutations and cytoplasmic TDP-43 accumulation. However, despite the similarity in binding of WT and mutant CHCHD10 to TDP-43, WT CHCHD10 overexpression maintained TDP-43 localization exclusively to the nucleus, suggesting that WT but not mutant CHCHD10 inhibits the exit of TDP-43 from the nucleus. This notion is supported by our finding that CHCHD10 depletion and FTD/ALS mutations produced the same TDP-43 phenotype, indicating that the binding of CHCHD10 mutants to TDP-43 *per se* is not the principle cause for cytoplasmic TDP-43 accumulation. Rather, we interpret these results to indicate that FTD/ALS-associated CHCHD10 mutations alter the activity of the protein to act in a dominant negative manner (Fig. 9). As an example, the S59L mutation was unable to effectively promote the mislocalization of TDP-43 in the absence of either N-terminal 16 or C-terminal 119–142 residues, indicating that the S59L mutation acts in concert with these domains for its dominant negative activity. Interestingly, both domains were required for efficient mitochondrial localization of CHCHD10. We observed that 35–45% of the cytoplasmically mislocalized TDP-43 by CHCHD10 depletion or expression of CHCHD10 mutations (R15L and S59L) was also co-localized with mitochondria. These findings are consistent with previous studies demonstrating that overexpression of TDP-43 induces mitochondrial/synaptic dysfunction, ALS-associated TDP-43 mutations localize more readily to mitochondria than its wild-type counterpart^10^,^11^, and blockade of TDP-43 mitochondrial targeting also blocks its neuronal toxicity^12^.

It has been reported that overexpression of CHCHD10 S59L mutation in Hela cells induces mitochondrial dysfunction but inhibits apoptosis^14^. However in this study, we found no evidence that either R15L or S59L mutation exerted anti-apoptotic properties. Instead, WT CHCHD10 protected HT22 cells from TDP-43-induced apoptosis, whereas R15L and S59L mutations synergized with the pro-apoptotic actions of TDP-43. This potential discrepancy might be explained by the differences in the cell lines used and/or the inclusion of a specific stressor in our study, TDP-43, which was not examined in the previous study^14^. Nevertheless, the CHCHD10-associated apoptotic phenotype in HT22 cells commensurately mirrored the observations of synaptic integrity (synaptophysin and drebrin) *in vivo*, indicating that mitochondrial dysfunction exerted by CHCHD10 mutations effectively renders synapses more vulnerable in brain. Our findings taken together, therefore, provide a novel molecular linkage between the mitochondrial/synaptic toxicity of CHCHD10 dysfunction and the pathological cytoplasmic accumulation of TDP-43.

## Methods

### DNA constructs and siRNA

Mouse hippocampus-derived HT22 (ref. 24) and NIH3T3 (ATCC, CRL-1658) and HEK293T (ATCC, CRL-3216) cells were maintained in DMEM containing 10% FBS and 1% penicillin-streptomycin. The wtTDP43tdTomatoHA construct was a gift from Zuoshang Xu (Addgene #28,205)^7^. The shRNA lentivirus plasmid targeting mouse CHCHD10 was purchased from Abm (# i035693a, Richmond, BC, Canada). To generate mammalian constructs expressing human Flag-CHCHD10 variants (WT, R15L, Δ1–15, Δ119–142), the CHCHD10 coding region was amplified by PCR using pyrococcus furiosus DNA polymerase (PFU) (Agilent, Santa Clara, CA, USA) and corresponding WT or mutated primers (Supplementary Table 2) from pCMV-Sport6-CHCHD10 (Image ID 5,747,937), subjected to restriction digest (HindIII+SalI), and inserted into p3x-Flag vector (cut with HindIII+SalI). The S59L mutant was amplified by PCR using PFU and the indicated primers (Supplementary Table 2) from pCMV-Sport6-CHCHD10, subjected to restriction digest (HindIII+BmgBI), and inserted into p3x-Flag-CHCHD10 (cut with HindIII+BmgBI). To clone CHCHD10 variants into the rAAV9 plasmid, p3x-Flag-CHCHD10 and its mutants were digested by SpeI and SalI, and cloned into pTR2-MCS-IG-hGFP vector at SpeI and XhoI cloning sites. TDP43-tomato-HA was cloned into pTR12.1-MCSW vector using NheI (destroyed after ligation) and XhoI restriction sites. RFP was digested by XmalI and HpaI, and cloned into pTR12.1-MCSW vector using AgeI and EcoRV (destroyed after ligation) restriction sites. All cloned or subcloned plasmids were confirmed by sequencing before use in experiments. For transfections, cells were transfected for 24–48 h using lipofectamine 3000 according to the manufacturer's instructions (Invitrogen, Carlsbad, CA, USA). To transfect control and human CHCHD10 siRNA (5′-UGAAGCAGUGCAAGUACUA-3′, GE Dharmacon, Lafayette, CO, USA), 293T cells were transfected twice every 24 h. Cells were incubated 24–48 h for plasmid transfections and 72 h for siRNA transfections before harvest.

### Generation of CHCHD10 siRNA lentivirus

CHCHD10 shRNA lentivirus plasmid was purchased from Abm (# i035693a), with the target sequence corresponding to 5′-CAGCCGGGTCTTATGGCTCAGATGGCATC-3′. Lentivirus vectors were co-transfected with pVSVG and pAX2 using polyethylenimine in HEK293T cells overnight. Media was removed and replaced with serum-free media for 3 days, and conditioned media was collected and centrifuged to remove cell debris. The viral medium was filtered through syringe filter (0.45 μM), aliquoted and frozen in −80 °C.

### Primary neurons

Hippocampal and cortical neuron cultures were prepared from P0 mice^24^,^25^. In brief, both hippocampus and cortex were dissected separately in ice-cold HBSS and digested with trypsin. Mouse neurons were plated on glass coverslips or plates coated with poly-D-lysine (Sigma-Aldrich, St Louis, MO, USA) in neurobasal medium (Invitrogen), 2% glutamax, and 2% B27 supplement (Invitrogen).

### Antibodies and reagents

Antibodies to CHCHD10 (1:500, 25,671-1-AP, Proteintech, Rosement, IL, USA), M2 (1:1,000, F3165, Sigma-Aldrich), TOM20 (1:1,000, FL-145, Santa Cruz Biotechnology, Dallas, TX, USA), Drebrin (1:500, ab12,350, Abcam, Cambridge, MA, USA), Synaptophysin (1:500, S5768, Sigma-Aldrich), TDP-43 (1:500, MABN45, Merck Millipore, Darmstadt, Germany), LaminB1 (1:1,000, D4Q4Z, Cell Signaling, Danvers, MA, USA), Actin (1:3,000, A5316, Sigma-Aldrich), Actin (1:1,000, sc-1,616, Santa Cruz, Dallas, TX, USA), tRFP (1:1,000, AB233, Evrogen, Moscow, Russia), horseradish peroxidase-linked secondary antibodies (1:5,000, Jackson ImmunoResearch, West Grove, PA, USA), and fluorescently labelled secondary antibodies (1:1,000, Invitrogen) were obtained from the indicated sources.

### Cell lysis and immunoblotting

Cells were lysed using RIPA lysis buffer (50 mM Tris pH 7.4, 150 mM NaCl, 2 mM ethlenediaminetetraacetic acid, 1% NP-40, 0.1% sodium dodecyl sulfate). For co-immunoprecipitation experiments, cells were lysed in NP-40 lysis buffer (50 mM Tris pH 7.4, 150 mM NaCl, 2 mM ethlenediaminetetraacetic acid, 1% NP-40). Biochemical nuclear versus cytoplasmic isolation was performed using the nuclear isolation kit for cells (Thermo Scientific, Rockford, IL, USA) according to the manufacturer's instructions. For SDS-insoluble fractions, pellets from RIPA-insoluble lysates were extracted with 4 × lithium dodecyl sulfate (LDS) sample buffer followed by sonication and boiling. Total protein concentrations were quantified by a colorimetric detection assay (BCA Protein Assay, Pierce, Rockford, IL, USA). Equal amounts of protein lysates were separated by SDS–polyacrylamide gel electrophoresis, and transferred to Immobilon-P membranes (Merck Millipore, Darmstadt, Germany). Proteins of interest were probed by primary antibodies and corresponding peroxidase-conjugated secondary antibodies, followed by detection by ECL (Merck Millipore, Darmstadt, Germany) and capture using the LAS-4000 imager (GE Healthcare Biosciences, Pittsburgh, PA, USA). Images have been cropped for presentation. Full-length blot images with molecular weight markers are presented in Supplementary Figs 8–12.

### Quantitative real-time RT–PCR

Quantitative real-time RT–PCR was performed using Roche LightCycler 96 System (Life Science, San Francisco, CA, USA), following the manufacturer's recommended conditions. Total RNA was isolated from transiently transfected cells using Trizol reagent (Invitrogen), reverse transcribed (Superscript III, Invitrogen), and subjected to quantitative PCR analysis using Syber green master mix (Invitrogen). The comparative threshold cycle (Ct) value was used to calculate the amplification factor, and the relative amount of targets was normalized to GAPDH levels^39^. The primer sequences for each target transcript are provided in Supplementary Tables 3 and 4.

### Mitochondrial health and apoptosis assays

HT22 cells were plated onto 35-mm glass bottom dishes or 24-well plates with glass coverslips (both coated with fibronectin). After 24 h, cells were either transduced with lentivirus or transfected with indicated plasmids for up to 48 h. For Annexin V (BD Biosciences, San Jose, CA, USA), JC-1 (Invitrogen), and MitoSox-Red (Invitrogen) staining, live cells were washed with PBS and stained with Annexin V-FITC, JC-1, MitoSox-Red for 20 min. Cells were washed one time with binding buffer, and images were captured by Olympus FV10i confocal microscope (Tokyo, Japan)^24^. AlamarBlue cell viability assays (Invitrogen) were performed per the manufacturer's instructions in a microtiter plate reader with absorbance reading at 570 nm, using 600 nm as a reference wavelength for normalization.

### Immunocytochemistry and Immunohistochemistry

For immunocytochemistry (ICC), cells were fixed using 4% paraformaldehyde for 15 min at room temperature. Cells were blocked using 3% BSA with 0.1% Triton-x-100 for 1 h and subjected in primary antibodies incubation for overnight at 4 °C and secondary antibody (Alexa-405, Alexa-488, or Alexa-594, Invitrogen) incubation for 1 h at room temperature, followed by counterstaining with DAPI and mounting. For immunohistochemistry (IHC), mice were perfused with PBS, and half brains were immediately stored at −80 °C for biochemical analysis, and the other half was fixed with 4% paraformaldehyde at 4 °C for 24 h followed by cryoprotection in 30% sucrose. Thirty-micron sections were blocked using normal goat serum for 1 h and subjected to primary antibodies (anti-drebrin 1:100 Abcam, Cambridge, MA, USA; or anti-synaptophysin 1:100, Sigma) at 4 °C for overnight, followed by secondary antibody (Alexa-405 and Alexa-488) incubation for 1 h at room temperature before mounting. Images were captured with the Olympus FV10i confocal microscope, and the immunoreactivities were quantified from hippocampus CA3 region using the Image J software (National Institutes of Health, Bethesda, MD, USA). Immunoreactivities were quantitated from every 12th serial section through an entire hippocampus. In ICC and IHC experiments, all comparison images were acquired with identical laser intensity, exposure time, and filter. Adjustments to the brightness/contrast were applied equally to all comparison images. Regions of interest were chosen randomly, and investigators were blinded to experimental conditions during image acquisition and quantification.

### Generation of rAAV9 and stereotaxic injections in mice

Recombinant AAV9 viruses were generated by co-transfection of serotype vector expressing the interest gene with pAAV9 and pXX6 in HEK293 cells and subjected to purification as described^40^. C57BL6 mice were supplied with water and food *ad libitum* with 12-hour light/dark cycle at standard vivarium conditions. For brain injections, isoflurane anaesthetised mice (2-month old, equally balanced for gender per condition) were placed onto the stereotaxic apparatus, and burr holes were drilled using a dental drill bit (SSW HP-3; SSWhite Burs, Lakewood, NJ, USA) through the cranium. Bilateral injections were made into the hippocampus with a 26 gauge needle attached to a 10-μl syringe (Hamilton, Reno, NV, USA) at the following coordinates: anteroposterior −2.7 mm, lateral −2.7 mm, and vertical 3.0 mm. A total volume of 2 μl purified rAAV9 (1 × 10^12^ vg ml^−1^) was injected over a 2 min period using the convection enhanced delivery method^40^. The incision was then cleaned and closed with surgical sutures. The mice recovered within 20 min and were housed singly until the time they were sacrificed 4 weeks post injection.

### *C. elegans* transgenesis

To generate the BFP fusion CHCHD10 variants, Multisite Gateway cloning entry vectors containing promoter *eef-1A.1* (p*eef-1A.1*), full-length CHCHD10 variants (wild-type, R15L and S59L) with stop codon removed, and tagBFP::*unc-54* 3′-untranslated region (UTR) were amplified via Phusion High-Fidelity DNA Polymerase (NEB) and cloned into pDONRP4-P1r, pDONR221 and pDONRP2R-P3, respectively. To generate *att*B4/*att*B1r flanked p*eef-1A.1,* Gateway-compatible primers were used to amplify p*eef-1A.1* from pCFJ601 (Addgene). *att* flanked CHCHD10 variants were subcloned from p3x-Flag-CHCHD10 variants (WT, R15L and S59L) before BP cloning. Lastly, to specifically generate ‘tagBFP::*unc-54* 3′-UTR', 24 base pair overlapping PCR products of tagBFP amplified from pSR04 (Addgene) and *unc-54* 3′-UTR amplified from pCFJ104 (Addgene) were generated and PCR stitching was used to fuse the *att* flanked product before BP cloning^41^. Three expression clones were generated containing p*eef-1A.1*, CHCHD10 (wild-type, R15L or S59L), and tagBFP::*unc-54* 3′-UTR by LR reaction with pCG150 (Addgene). The three resulting expression constructs were fully sequence-verified before microinjection.

### *C. elegans* strains and microinjections

Unless otherwise stated, the *C. elegans* strains used were obtained from the Caenorhabditis Genetics Center (University of Minnesota): wild-type Bristol N2, *har-1*(*gk3124*) III, and *dvIs62* X. The *har-1*(*gk3124*) strain was provided by the *C. elegans* Gene Knockout Project at the Oklahoma Medical Research Foundation, which was part of the International *C. elegans* Gene Knockout Consortium. Transgenic strains used in this study including CHCHD10 variants (WT, R15L and S59L) and their corresponding CGC designations are listed in Supplementary table 1. Strains were maintained at 16 °C on nematode growth media (NGM) agar plates with OP50 *Escherichia coli* unless otherwise indicated. Experimental plates were age-synchronized using timed egg laying^42^. Transgenic *C. elegans* strains expressing wild-type or mutant hCHCHD10 variants were generated by microinjection of *har-1* knockout animals, *har-1*(*gk3124*) III, with p*eef-1A.1*::CHCHD10 (wild-type, R15L or S59L)::BFP::*unc-54* 3′-UTR (50 ng μl^−1^), and BFP positive animals were selected for at least three generations before experimentation. All microinjections were performed by Knudra Transgenics (Murray, UT, USA). Multiple lines per transgene were generated and propagated separately. All strains are available upon request.

### *C. elegans* locomotion assays

*Motility*. Age-synchronized worms per strain (3 day adults) were transferred to a fresh NGM plate, exposed to room temperature for 30 min, and then videotaped for 1 min at room temperature. Worm motility (body lengths per second, BLPS) was measured using the ImageJ Plugin wrMTrck (Jesper Sondergaard Pedersen)^43^.

*Thrashing*. Age-synchronized worms per strain (3 day adults) were transferred to room temperature M9 buffer and recorded 2 min after introduction to liquid for 1 min. Thrashing speed (BBPS) was measured manually with a click counter. Per cent time spent curling was measured manually as the number of frames spent curled per total number of frames by a blinded investigator.

### *C. elegans* mitosox-red imaging

Age-synchronized worms per strain (3 day adults) were transferred to fresh NGM plates without *E. coli* and allowed to crawl on plates for 15 min at 16 °C to clear their gut. Afterwards, worms were incubated in M9 Buffer with 10 mM sodium azide and 5 μM mitosox-red (Invitrogen) for 30 min at room temperature, mounted live on agar pads, and imaged immediately with an Olympus FV10i confocal microscope. Average intensity of head region excluding mouth, pharynx and intestine was quantified with ImageJ (NIH ImageJ, Bethesda, MD, USA) in a blinded fashion.

### *C. elegans* lifespan assay

L4 stage age-synchronized worms per strain were transferred to OP50 plates and kept at 16 °C. Number of dead worms per plate was scored each day by a blinded observer. Worms were assumed dead if they remained motionless after prodding with an eyebrow pick. Living worms were transferred to fresh plates every 2 days until there were no surviving worms.

### *C. elegans* immunoblotting

Well-fed worms were washed three times with M9 buffer and then lysed using repeated freeze-thaw and sonication in a lysis buffer (50 mM Tris-Cl, 150 mM NaCl, 2 mM EDTA, and 1% Triton X-100 supplemented with protease and phosphatase inhibitor cocktails) and centrifuged at 130,000 *g* for 5 min. Protein quantification of the soluble sample was performed by a colorimetric detection reagent (BCA protein assay, Pierce). Equal amounts of protein were subjected to SDS–polyacrylamide gel electrophoresis and transferred to nitrocellulose membranes for immunoblotting. After probing with primary antibody (anti-actin (I-19), Santa Cruz, Dallas, TX, USA, anti-CHCHD10 Proteintech; anti-tRFP Evrogen), the corresponding peroxidase-conjugated secondary antibody was detected by ECL western blot reagents (Pierce). ECL images were captured by the Fuji/GE LAS-4,000 imager (LAS-4,000, Pittsburgh, PA, USA) and quantified using ImageJ software (NIH ImageJ).

### Statistical analysis and graphs

Statistical data were analysed by GraphPad Prism 6.0 software (GraphPad Software, San Diego, CA, USA) using Student's *t*-test, one- or two-way analysis of variance. One- or two-way analysis of variance was followed by the indicated *post hoc* tests. Survival analysis was performed by the Log-rank (Mantel–Cox) test. All quantitative graphs with error bars were expressed as mean±s.e.m.

### Data availability

The data that support the findings of this study are available from the corresponding authors on reasonable request.

## Additional information

**How to cite this article:** Woo, J.-A. A. *et al*. Loss of function *CHCHD10* mutations in cytoplasmic TDP-43 accumulation and synaptic integrity. *Nat. Commun.* **8,** 15558 doi: 10.1038/ncomms15558 (2017).

**Publisher's note:** Springer Nature remains neutral with regard to jurisdictional claims in published maps and institutional affiliations.

## Supplementary Material

Supplementary Information Supplementary Figures and Supplementary Tables.

Supplementary Movie 1 A representative movie showing normal liquid thrashing behavior of N2 control *C. elegans*.

Supplementary Movie 2 A representative movie showing abnormal curling behavior of the *har1-/- strain* while thrashing in liquid.
