# Supplementary material for: Loss of function CHCHD10 mutations in cytoplasmic TDP-43 accumulation and synaptic integrity
Source: Nat Commun. 2017 Jun 6;8:15558. doi: 10.1038/ncomms15558 (PMC5467170; doi:10.1038/ncomms15558)
Supplement: Supplementary Information — Supplementary Figures and Supplementary Tables. [file ncomms15558-s1.pdf]

Supplementary Fig. 1

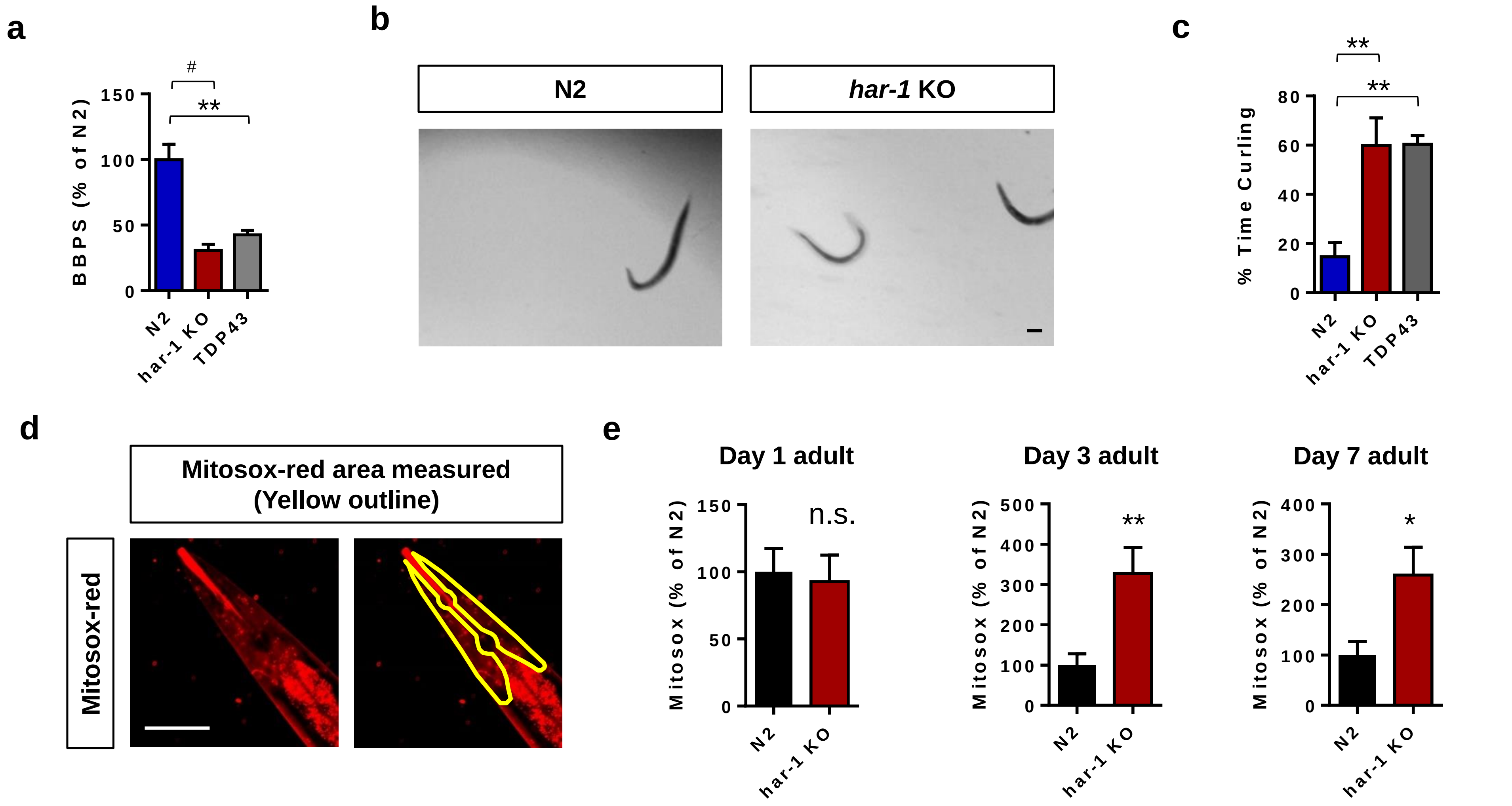

Supplementary Figure 1 | Loss of *har-1* or TDP-43 expression alters thrashing and curling behavior in *C. elegans*. (**a**) Liquid thrashing rate (body bends per second, BBPS) measured in M9 buffer after 30 min at 30°C and normalized to N2 control (1-way ANOVA, posthoc Tukey, **p<0.01, $^{\#}$p<0.001, n=5 worms/genotype). (**b,c**) Age-synchronized N2, *har-1* KO, and TDP43 worms transferred to fresh M9 buffer and videotaped to measure curling behavior. Scale bar, 100μm. (**a**) Representative video of N2 and *har-1* KO worms, demonstrating abnormal curling behavior in *har-1* KO worms at ambient room temperature (22°C). (**c**) Percent time spent curling measured after 30 min at 30°C (1-way ANOVA, posthoc Tukey, **p<0.01, n=5 worms/genotype). (**d**) Representative mitosox-red images demarcating the region of mitosox-red measurement and quantification outlined in yellow (head region excluding mouth, pharynx, and intestine). Scale bar, 100μm. (**e**) Age-synchronized worms (adult days 1, 3, & 7) at 22°C stained with 5 μM mitosox-red in M9 buffer for 30 minutes, mounted live on agar pads, imaged by confocal microscopy, quantified with Image J (head region excluding mouth, pharynx, and intestine), and normalized to N2 controls (1-way ANOVA, *p<0.05, **p<0.01, n=10-13 worms/strain).

Supplementary Fig. 2

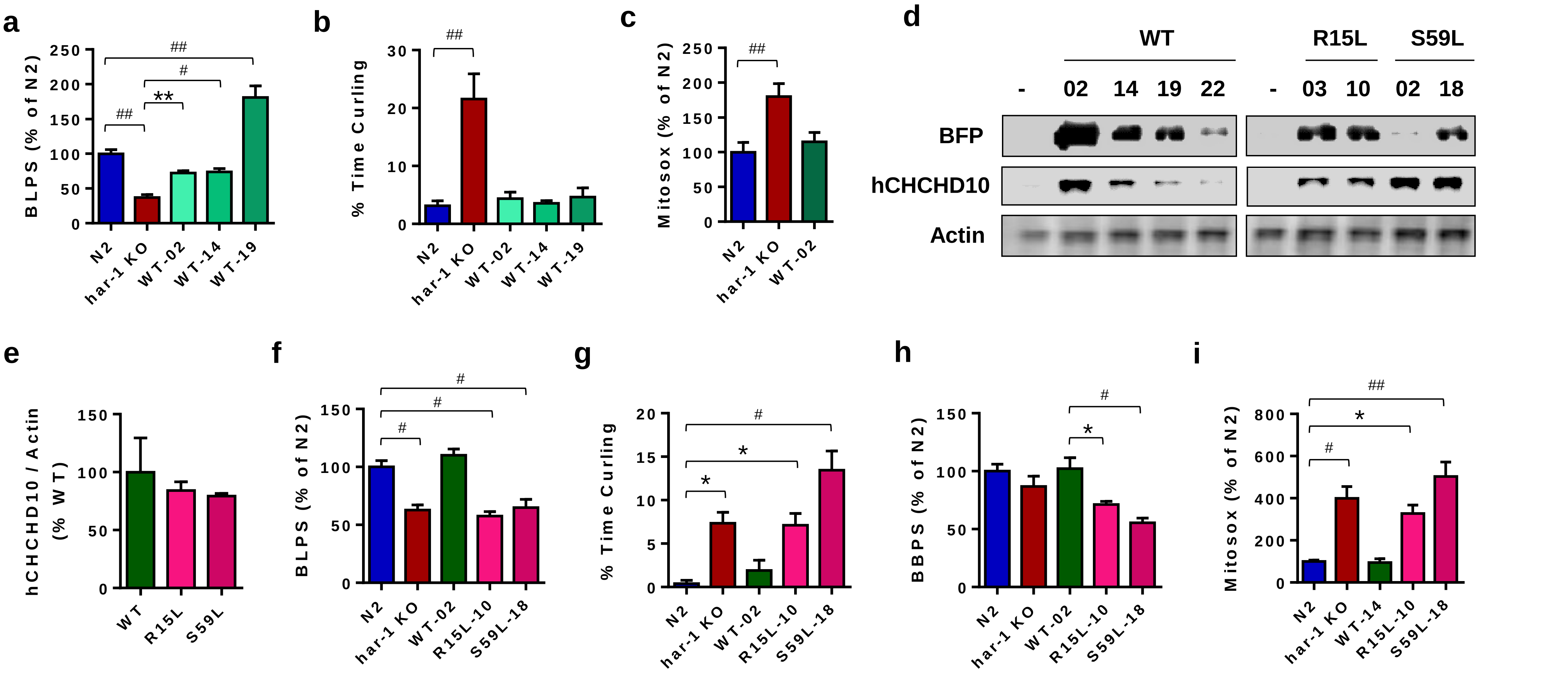

Supplementary Figure 2 | Wild type human CHCHD10 and FTD/ALS mutations in *har-1* KO *C. elegans*. (**a,b**) Age-synchronized N2, *har-1* KO, and *har-1* KO worms expressing WT CHCHD10 transferred to fresh NGM plate or M9 buffer and videotaped to measure motility and curling behavior. Motility (BLPS) and percent time spent curling measured at ambient room temperature (22°C) normalized to N2 controls (1-way ANOVA, posthoc Tukey, $^{**}p<0.01$, $^{\#}p<0.001$, $^{\#\#}p<0.0001$, n=5-12 worms/strain). (**c**) Age-synchronized worms at 22°C stained with 5 μM mitosox-red in M9 buffer, mounted live on agar pads, imaged by confocal microscopy, quantified with Image J (head region excluding mouth, pharynx, and intestine), and normalized to N2 controls (1-way ANOVA, $^{\#\#}p<0.0001$, n=5 worms/strain). (**d,e**) Immunoblotting of *har-1* KO and *har-1* KO worms expressing human CHCHD10-BFP variants (WT, R15L, & S59L) with BFP and CHCHD10 antibodies. (**e**) Quantification of hCHCHD10 variants relative to Actin (WT-02/14/19 vs. R15L-03/10 vs. S59L-02/18 lines). (**f-h**) Age-synchronized N2, *har-1* KO, and *har-1* KO worms expressing WT, R15L, or S59L CHCHD10 transferred to fresh NGM plate or M9 buffer and videotaped to measure motility (BLPS), thrashing (BBPS), and curling behavior at 22°C normalized to N2 controls (1-way ANOVA, posthoc Tukey, $^{*}p<0.05$, $^{**}p<0.01$, $^{\#}p<0.001$, $^{\#\#}p<0.0001$, n=5-10 worms/strain). (**i**) Age-synchronized day 3 adult worms at 22°C stained with 5 μM mitosox-red in M9 buffer for 30 minutes, mounted live on agar pads, imaged by confocal microscopy (head region excluding mouth, pharynx, and intestine), quantified with Image J, and normalized to N2 controls (1-way ANOVA, $^{*}p<0.05$, $^{\#}p<0.001$, $^{\#\#}p<0.0001$, n=5 worms/strain).

Supplementary Fig. 3

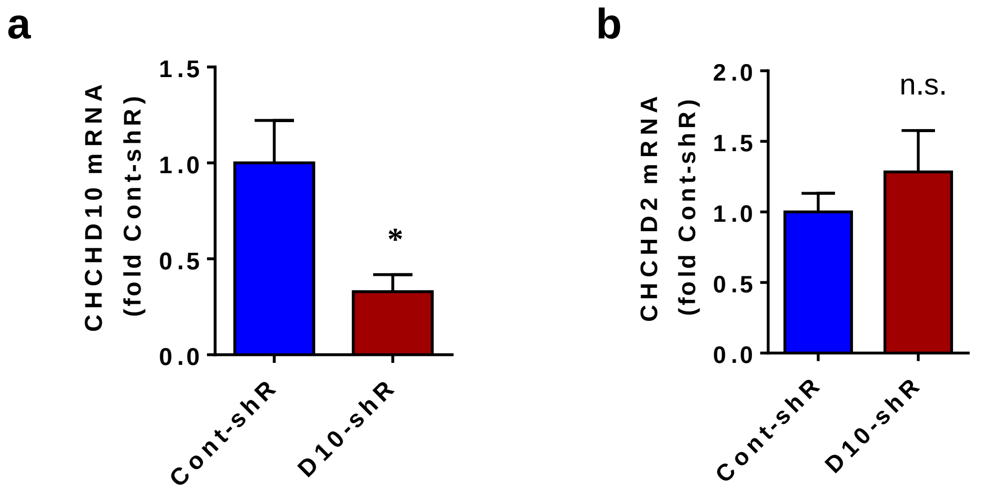

Supplementary Figure 3 | CHCHD10 knockdown does not alter CHCHD2 expression. (**a,b**) Mouse hippocampus-derived HT22 neuroblastoma cells transduced with control shRNA/GFP or CHCHD10 (D10) shRNA/GFP lentivirus and subjected to qRT-PCR for CHCHD10 and CHCHD2 and normalized to Cont-shR controls (t-test, *p<0.05, n=3 replicates each).

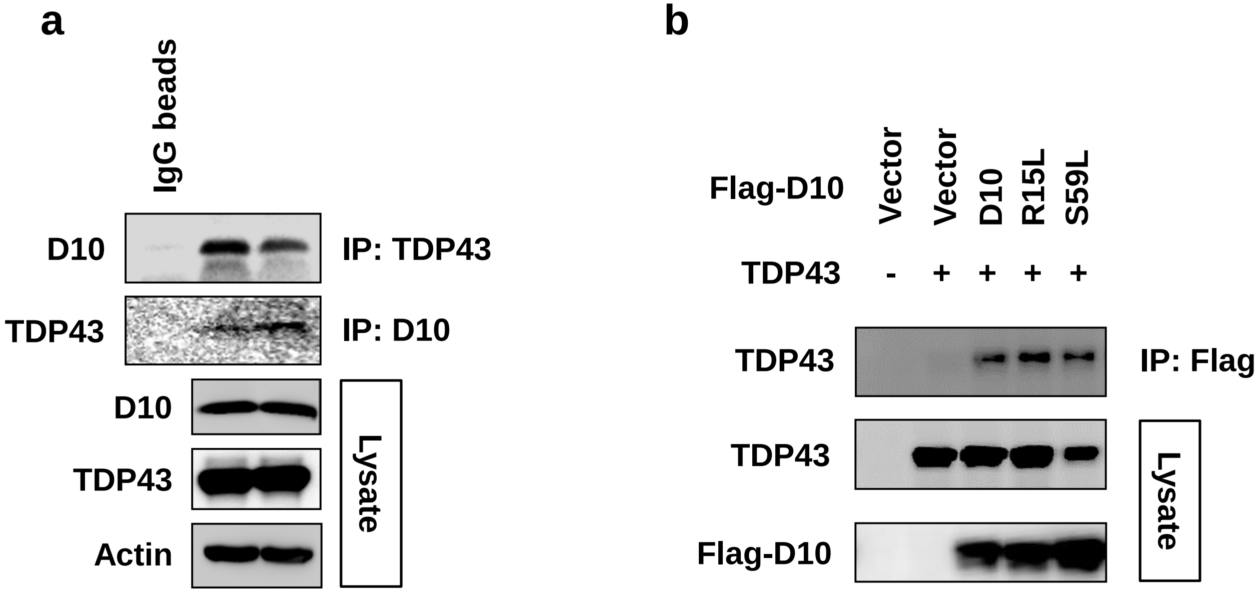

Supplementary Figure 4 | Endogenous and exogenous CHCHD10 forms complexes with TDP-43. (**a**) Lysates from HEK293T cells immunoprecipitated with IgG beads alone or with IgG beads plus antibodies to TDP-43 or CHCHD10 and immunoblotted for the indicated proteins. (**b**) HT22 cells transfected with/without TDP-43-tomato and the indicated Flag-CHCHD10 variants (WT, R15L, & S59L) and lysates subjected to co-IP with Flag (M2) antibody and/or immunoblotting for the indicated proteins.

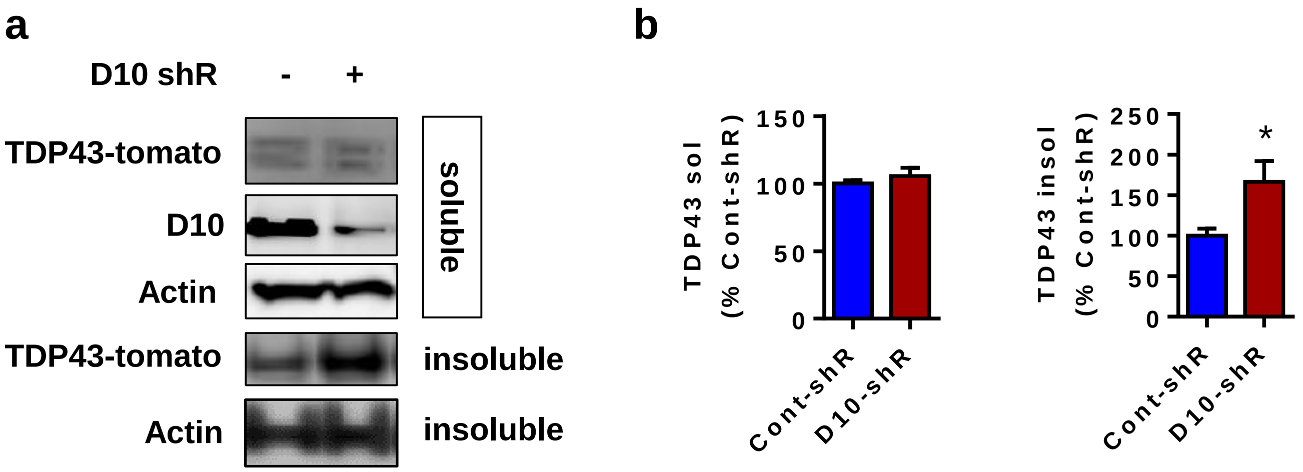

Supplementary Figure 5 | CHCHD10 knockdown increases SDS-insoluble TDP-43. (**a,b**) NIH3T3 cells transduced with control shRNA/GFP or CHCHD10 (D10) shRNA/GFP lentivirus and transfected with TDP-43-tomato, subjected to separation of SDS-soluble versus insoluble proteins, and immunoblotted for the indicated proteins. Representative blots shown. (**b**) Quantification of SDS-soluble versus insoluble TDP-43-tomato (t-test, *$p<0.05$, n=4 replicates).

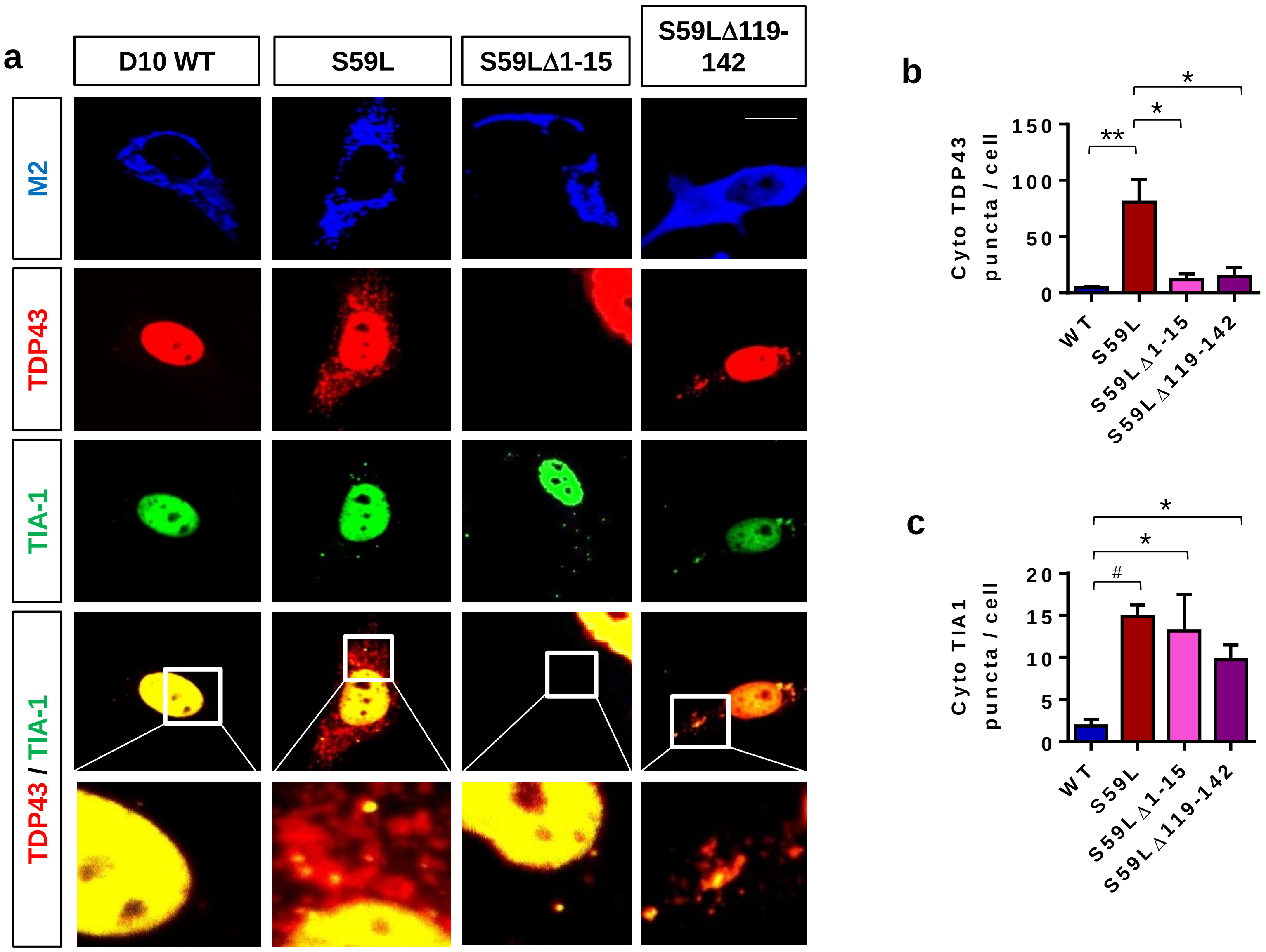

Supplementary Figure 6 | Deletion of N- or C-terminal domains of CHCHD10 mitigate S59L mutation-induced accumulation of cytoplasmic TDP-43 but not Tia-1. (**a-c**) NIH3T3 cells co-transfected with TDP43-tomato and the indicated Flag-CHCHD10 variants and subjected to ICC for Flag (M2), Tia-1, and direct fluorescence for TDP-43-tomato. White outlined boxes magnified in bottom panels (**a**). Scale bar, 10μm. (**b,c**) Quantification of cytoplasmic TDP-43-tomato puncta per cell and cytoplasmic Tia-1 puncta per cell (1-way ANOVA, posthoc Tukey, *p<0.05, **p<0.01, #p<0.001, n=6 replicates).

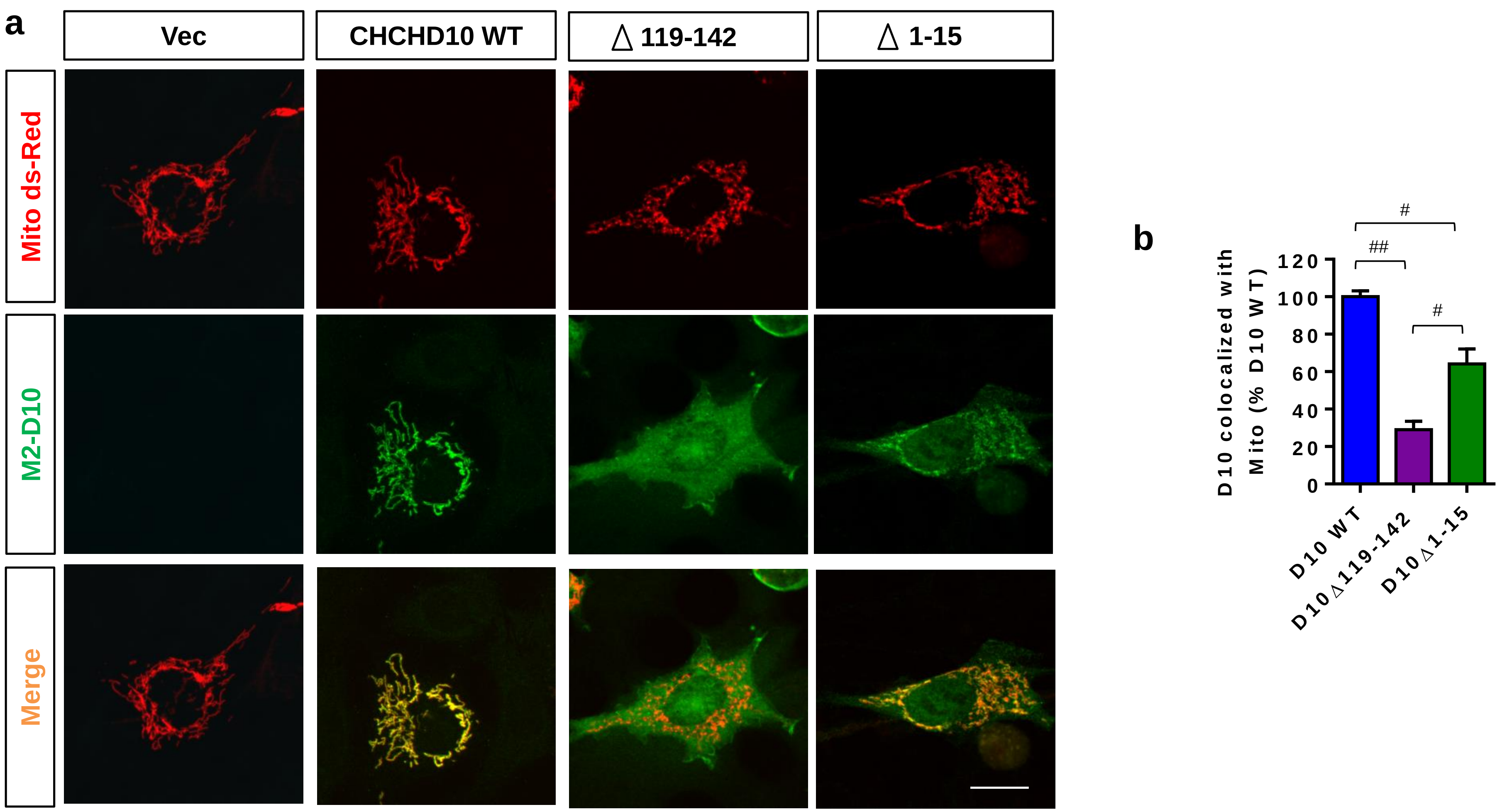

Supplementary Figure 7 | Deletion of N- or C-terminal domains alter the localization of CHCHD10. (**a,b**) NIH3T3 cells co-transfected with mito-dsRed and Flag-CHCHD10 variants (WT, Δ119-142, & Δ1-15) and subjected to ICC for Flag (M2) and direct visualization of mito-dsRed by confocal microscopy. Scale bar, 10µm. (**b**) Quantification of CHCHD10 colocalization with mito-dsRed by Image J (1-way ANOVA, posthoc Tukey, $^{\#}p<0.001$, $^{\#\#}p<0.0001$, n=6 replicates).

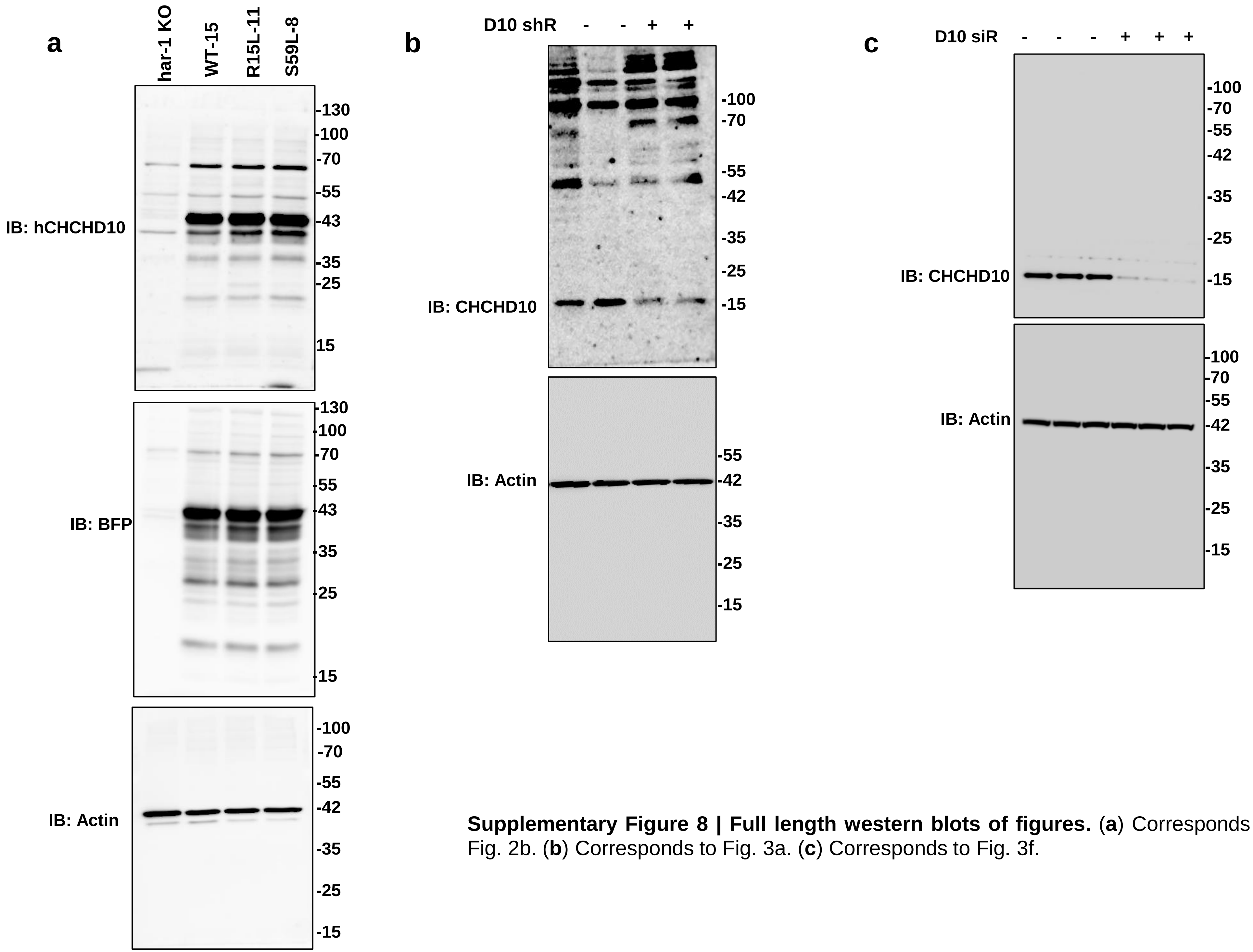

Supplementary Figure 8 | Full length western blots of figures. (**a**) Corresponds to Fig. 2b. (**b**) Corresponds to Fig. 3a. (**c**) Corresponds to Fig. 3f.

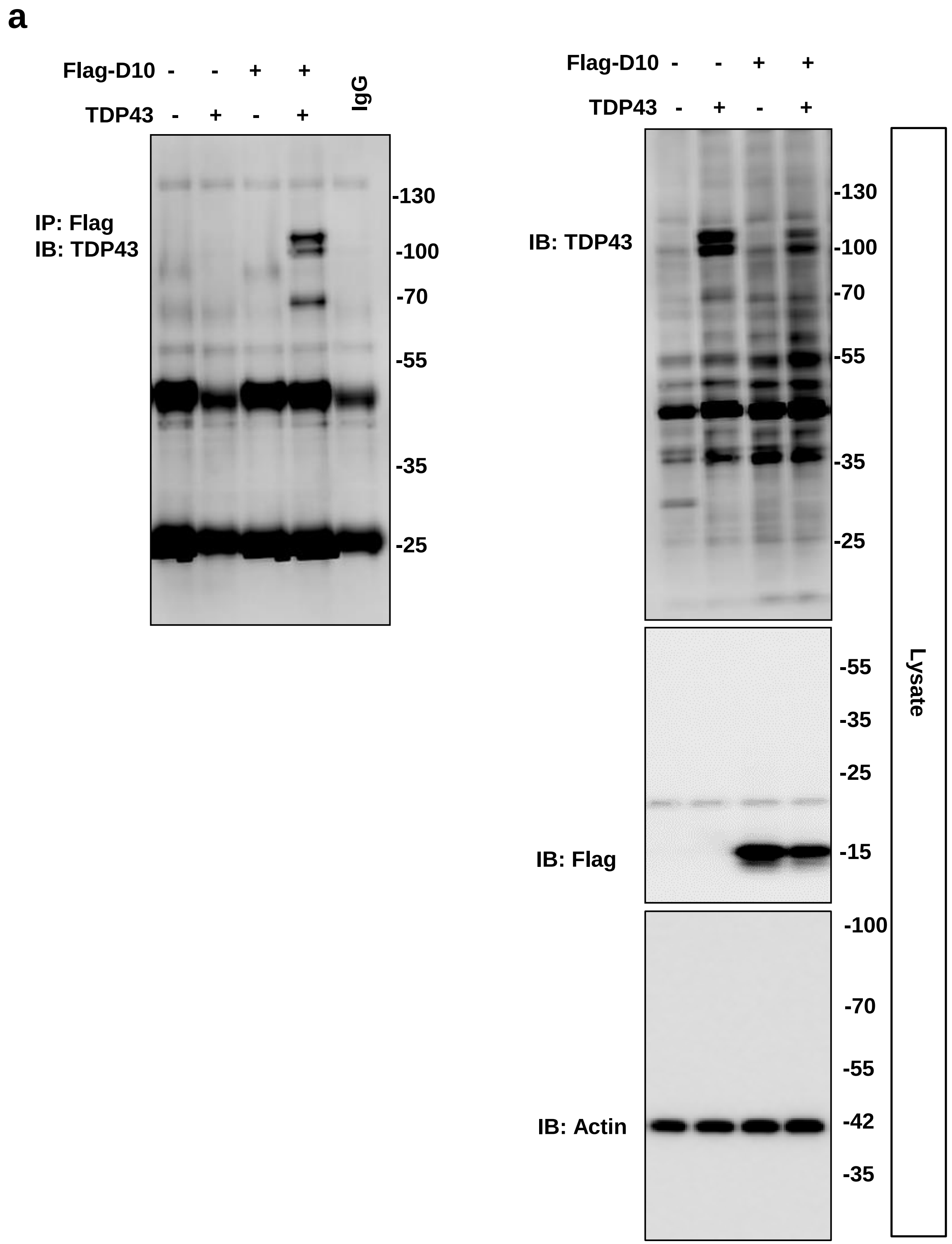

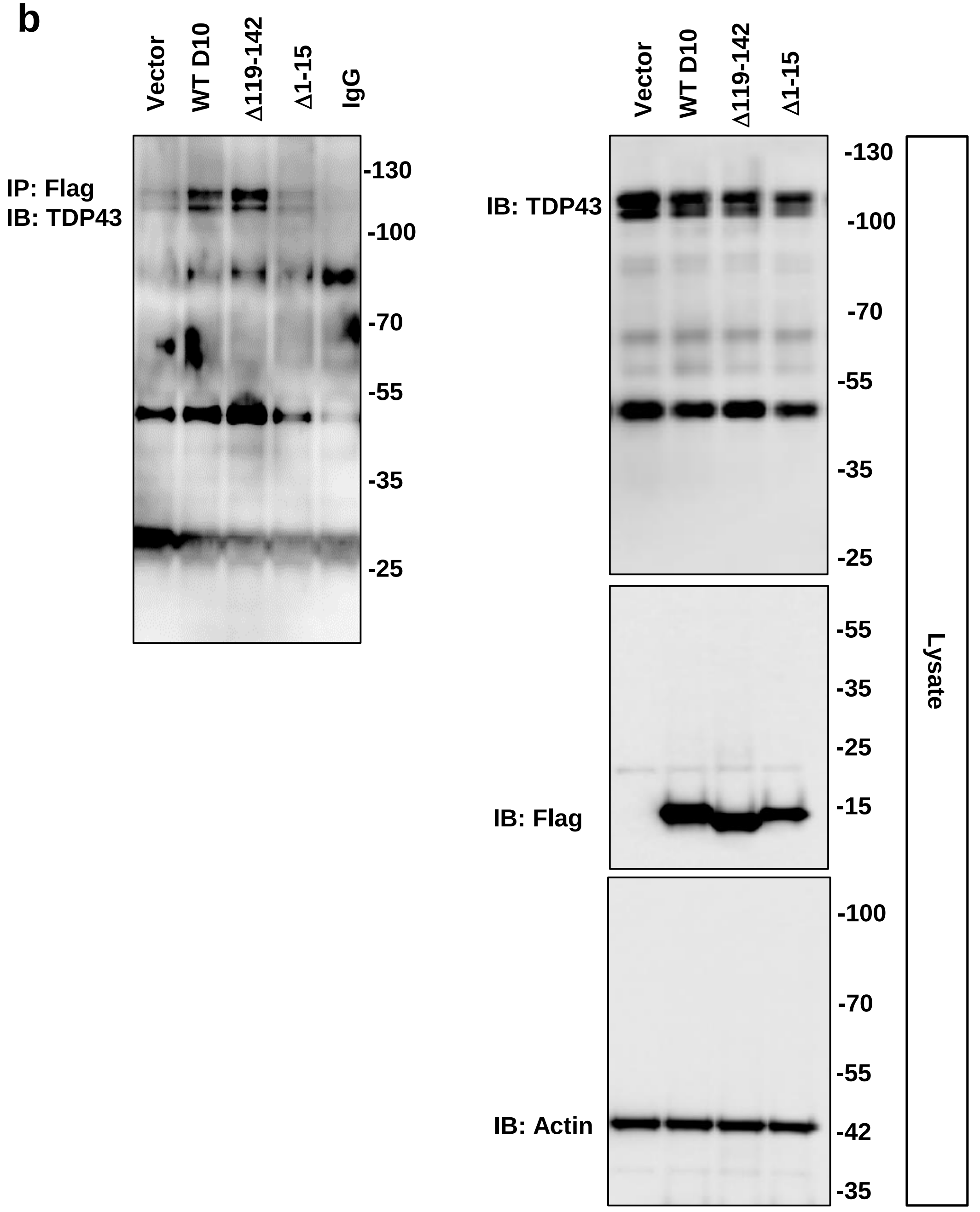

Supplementary Figure 9 | Full length western blots of figures. (**a**) Corresponds to Fig. 5a. (**b**) Corresponds to Fig. 5c.

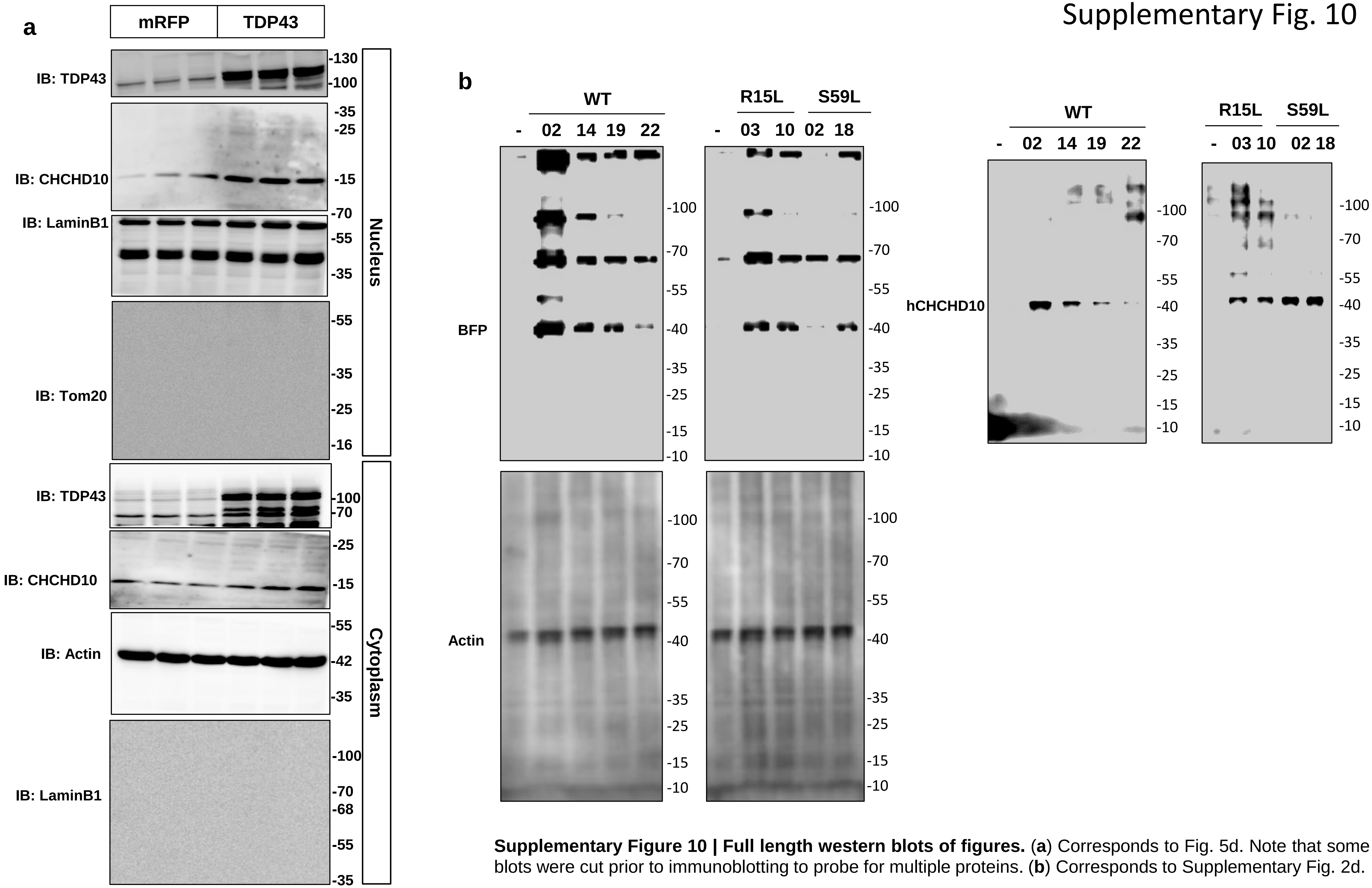

Supplementary Figure 10 | Full length western blots of figures. (**a**) Corresponds to Fig. 5d. Note that some blots were cut prior to immunoblotting to probe for multiple proteins. (**b**) Corresponds to Supplementary Fig. 2d.

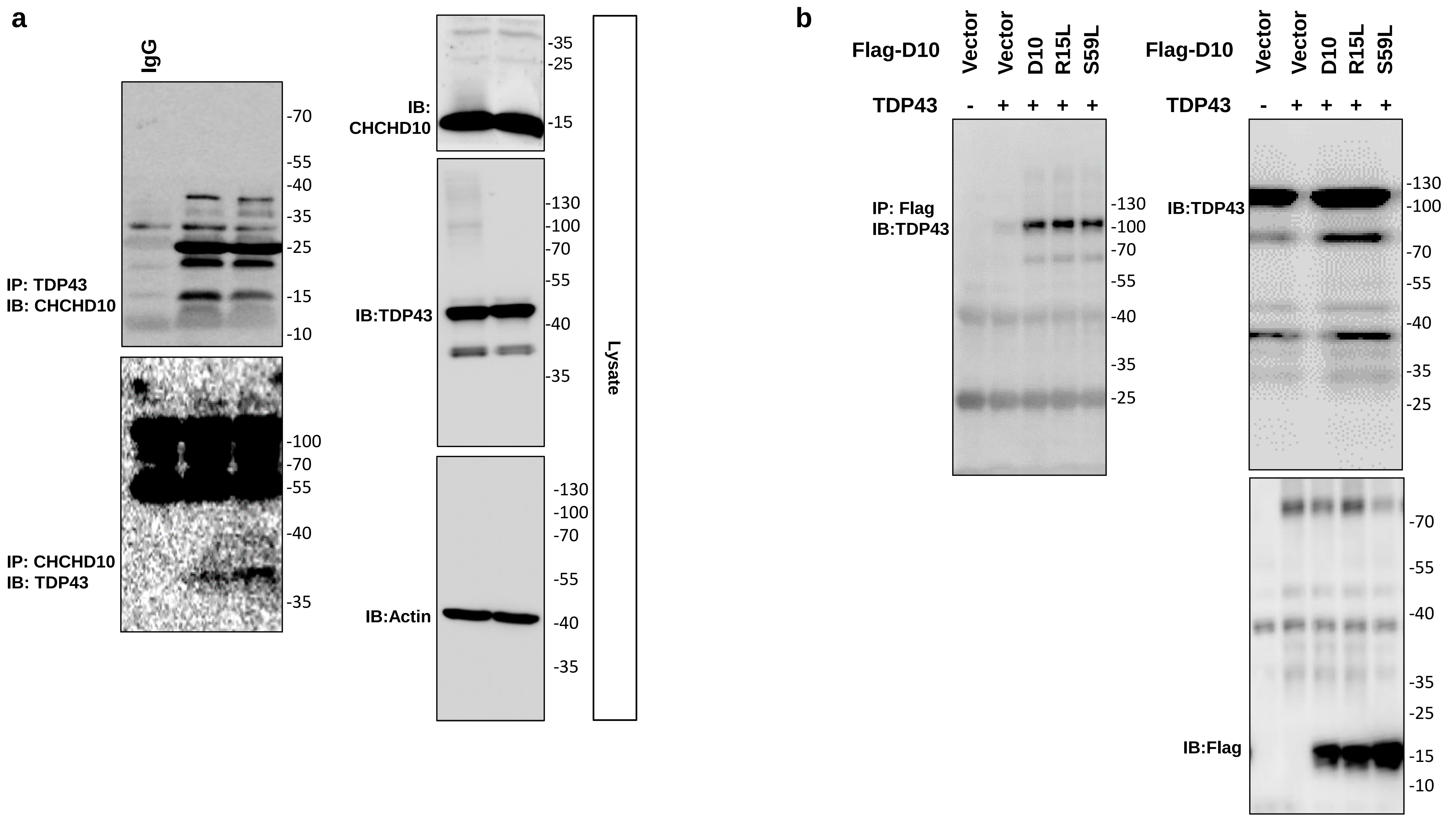

Supplementary Figure 11 | Full length western blots of figures. (**a**) Corresponds to Supplementary Fig. 4a. (**b**) Corresponds to Supplementary Fig. 4b.

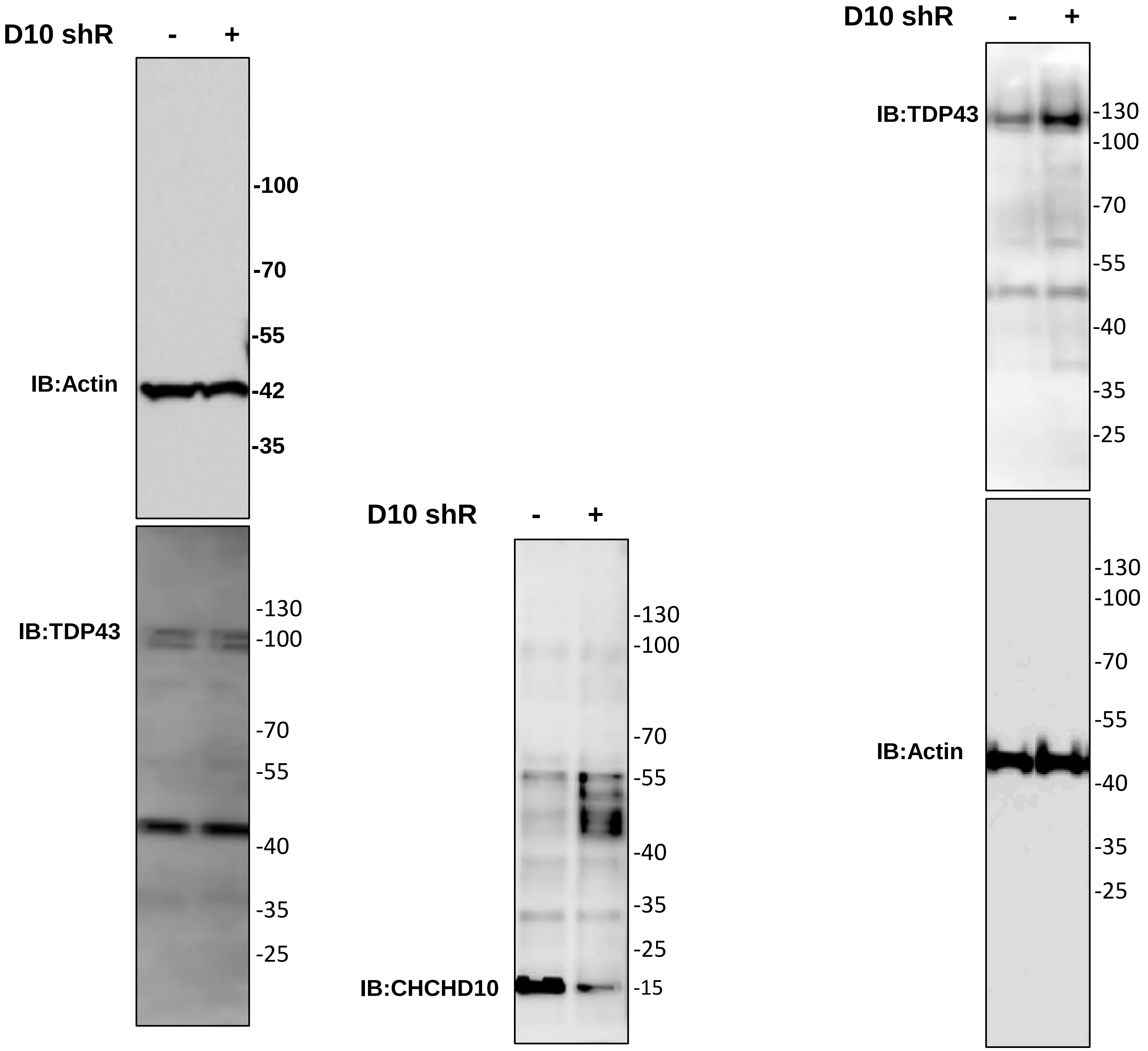

Supplementary Figure 12 | Full length western blots corresponding to Supplementary Fig. 5a.

Supplementary Table 1 *C. elegans* strain designations and genotypes

CGC Designation	Manuscript Designation	Genotype
N2	N2	wild type
VC3169	*har-1-/-* or *har-1* KO	*har-1*(gk3124)
CL6049	TDP43 transgenic	(dvIs62 [*snb-1*p::hTDP-43/3' long UTR + *mtl-2*p::GFP] X)
SDW008	*har1-/-*;CHCHD10 WT-02	*har-1*(gk3124); (asdEx006[*eef-1A.1*p::CHCHD10(wt)::BFP::*unc-54* 3'UTR]
SDW009	*har1-/-*;CHCHD10 WT-14	*har-1*(gk3124); (asdEx007[*eef-1A.1*p::CHCHD10(wt)::BFP::*unc-54* 3'UTR]
SDW010	*har1-/-*;CHCHD10 WT-19	*har-1*(gk3124); (asdEx008[*eef-1A.1*p::CHCHD10(wt)::BFP::*unc-54* 3'UTR]
SDW011	*har1-/-*;CHCHD10 R15L-10	*har-1*(gk3124); (asdEx009[*eef-1A.1*p::CHCHD10(R15L)::BFP::*unc-54* 3'UTR]
SDW012	*har1-/-*;CHCHD10 S59L-18	*har-1*(gk3124); (asdEx010[*eef-1A.1*p::CHCHD10(S59L)::BFP::*unc-54* 3'UTR]
SDW013	*har1-/-*; CHCHD10 R15L-03	*har-1*(gk3124); (asdEx011[*eef-1A.1*p::CHCHD10(R15L)::BFP::*unc-54* 3'UTR]
SDW014	*har1-/-*;CHCHD10 S59L-02	*har-1*(gk3124); (asdEx012[*eef-1A.1*p::CHCHD10(S59L)::BFP::*unc-54* 3'UTR]
DEK001	*har1-/-*;CHCHD10 WT-15	*har-1*(gk3124); (asdEx013[*eef-1A.1*p::CHCHD10(wt)::BFP::*unc-54* 3'UTR]
DEK002	*har1-/-*;CHCHD10 R15L-11	*har-1*(gk3124); (asdEx014[*eef-1A.1*p::CHCHD10(R15L)::BFP::*unc-54* 3'UTR]
DEK003	*har1-/-*;CHCHD10 S59L-18	*har-1*(gk3124); (asdEx015[*eef-1A.1*p::CHCHD10(S59L)::BFP::*unc-54* 3'UTR]

Supplementary Table 2 Primers used to PCR clone p3x-Flag-CHCHD10 variants

Variant	Primer Sequence
CHCHD10-WT	Forward: 5' – TTTTT AAGCTT ATG CCT CGG GGA AGC CGC AGC G -3'<br>Reverse: 5' – TTTTT G TCGAC TCA GGG CAG GGA GCT CAG ACC GTA -3'
CHCHD10-R15L	Forward: 5' – TTTTT A AGCTT ATG CCT CGG GGA AGC CGC AGC GCG GCC TCC CGG CCA GCC AGC CTC CCA G -3'<br>Reverse: 5' – TTTTT G TCGAC TCA GGG CAG GGA GCT CAG ACC GTA -3'
CHCHD10-S59L	Forward: 5' – TTTTT AAGCTT ATG CCT CGG GGA AGC CGC AGC G -3'<br>Reverse: 5' – C CAT GAC GTG TCC CAC AGC CAA GCC CA -3'
CHCHD10-Δ1-15	Forward: 5' – TTTTT AAGCTT ATG GCC GCG CCC TCT GCC CAC CCG C-3'<br>Reverse: 5' – TTTTT G TCGAC TCA GGG CAG GGA GCT CAG ACC GTA -3'
CHCHD10-Δ119-142	Forward: 5' – TTTTT AAGCTT ATG CCT CGG GGA AGC CGC AGC G -3'<br>Reverse: 5'- TTTTT GTC GAC TCA GTC ACT CTG AGT GGT GGA ACA - 3'

Supplementary Table 3 Primers used for real-time qRT-PCR of mitochondria-encoded transcripts

Gene	Primer Sequence
ND1	ND1 Fwd 5’-ATGGCCAACCTCCTACTCCTCATT-3’<br>ND1 Rev 5’-TTATGGCGTCAGCGAAGGGTTGTA-3’
ND2	ND2 Fwd 5’-ACTGCGCTAAGCTCGCACTGATTT-3’<br>ND2 Rev 5’-GATTATGGATGCGGTTGCTTGCGT-3’
ND3	ND3 Fwd 5’-CCCTACCATGAGCCCTACAAACAA-3’<br>ND3 Rev 5’-AGTCACTCATAGGCCAGACTTAGG-3’
ND4L	ND4L Fwd 5’-TATCGCTCACACCTCATATCCTCCCT-3’<br>ND4L Rev 5’-AGGCGGCAAAGACTAGTATGGCAA-3’
ND4	ND4 Fwd 5’-ACAAGCTCCATCTGCCTACGACAA-3’<br>ND4 Rev 5’-TTATGAGAATGACTGCGCCGGTGA-3’
ND5	ND5 Fwd 5’-ATCGGTTTCATCCTCGCCTTAGCA-3’<br>ND5 Rev 5’-ACCTAATTGGGCTGATTTGCCTGC-3’
ND6	ND6 Fwd 5’-AGGATTGGTGCTGTGGGTGAAAGA-3’<br>ND6 Rev 5’-ATAGGATCCTCCCGAATCAACCCT-3’
Cox I	Cox I Fwd 5’-ACCCTAGACCAAACCTACGCCAAA-3’<br>Cox I Rev 5’-TAGGCCGAGAAAGTGTTGTGGGAA-3’
Cox II	CoxII Fwd 5’-ACAGATGCAATTCCCGGACGTCTA-3’<br>CoxII Rev 5’-GGCATGAAACTGTGGTTTGCTCCA-3’
Cox III	CoxIII Fwd 5’-ACTTCCACTCCATAACGCTCCTCA-3’<br>CoxIII Rev 5’-TGGCCTTGGTATGTGCTTTCTCGT-3’
ATP6	ATP6 Fwd 5’-TAGCCCACTTCTTACCACAAGGCA-3’<br>ATP6 Rev 5’-TGAGTAGGTGGCCTGCAGTAATGT-3’
ATP8	ATP8 Fwd 5’-ACCGTATGGCCCACCATAATTACC-3’<br>ATP8 Rev 5’-TTTATGGGCTTTGGTGAGGGAGGT-3’
12S	12S Fwd 5’-AAACTGCTCGCCAGAACACTACGA-3’<br>12S Rev 5’-TGAGCAAGAGGTGGTGAGGTTGAT-3’
16S	16S Fwd 5’-TGTATGAATGGCTCCACGAGGGTT-3’<br>16S Rev 5’-TAGGGTCTTCTCGTCTTGCTGTGT-3’
CytB	CytB Fwd 5’-TCCTCCCGTGAGGCCAAATATCAT-3’<br>CytB Rev 5’-AAAGAATCGTGTGAGGGTGGGACT-3’

Supplementary Table 4 Primers used for real-time qRT-PCR of nuclear-encoded transcripts

Gene	Primer Sequence
Human NDUFS3	Fwd 5′-GTC AGA CCA CGG AAT GAT GTG-3′<br>Rev 5′-CTC AAA ACG GTT TTG CCG AG-3′
Human NDUFB6	Fwd 5′-CTG CAG CAG CTG CGA GA-3′<br>Rev 5′-GAA TAA TCC AGA CAG GTA CAA G-3′
Human COX4-2	Fwd: 5’-GCTATGCCCAGCGCTACTAC-3’<br>Rev: 5’-CATCTCCGCAAAGGTCTCAT-3’
Human GAPDH	Fwd. 5′-AAGGTCGGAGTCAACGGATT-3′<br>Rev. 5′-CCATGGGTGGAATCATATTGG-3′
Human ATG7	Fwd. 5′-ACCCAGAAGAAGCTGAACGA-3′<br>Rev.5′-AGACAGAGGGCAGGATAGCA-3′;
Mouse CHCHD10	Fwd 5’-GCCCATTTCACCTGACTCTAA-3’<br>Rev 5’-GGATGCCATCTGAGCCATAA-3’
Mouse CHCHD2	Fwd 5’-ATGGCCCAGATGGCTACC-3’<br>Rev 5’-CTGGTTCTGAGCACACTCCA-3’
